# Santin and cirsimaritin from *Betula pubescens* and *Betula pendula* buds induce apoptosis in human digestive system cancer cells

**DOI:** 10.1111/jcmm.17031

**Published:** 2021-11-09

**Authors:** Lukasz Szoka, Jolanta Nazaruk, Marcin Stocki, Valery Isidorov

**Affiliations:** ^1^ Department of Medicinal Chemistry Medical University of Bialystok Białystok Poland; ^2^ Department of Pharmacognosy Medical University of Bialystok Białystok Poland; ^3^ Institute of Forest Sciences Białystok University of Technology Białystok Poland

**Keywords:** apoptosis, *Betula*, buds, cirsimaritin, flavonoids, santin

## Abstract

Flavonoids are bioactive secondary metabolites of plants, which exert anti‐cancer effects. However, metabolism in enterocytes and the liver can influence the biological activity of flavonoids contained in the diet. Therefore, results from in vitro studies on cancer cells from the digestive tract and liver may reflect the real effects in the human body. Previously, we have found that the extract from birch buds exerts antiproliferative activity in a panel of cancer cells. In the present study, the anti‐cancer activity of ten flavonoids isolated from the buds of *Betula pubescens* and *Betula pendula* was characterized. Among them, santin and cirsimaritin significantly reduced viability, proliferation and clonogenicity of gastric (AGS), colon (DLD‐1) and liver (HepG2) cancer cells. Both flavonoids induced apoptosis, accompanied by activation of caspase‐3, caspase‐7, caspase‐8 and caspase‐9. Moreover, upregulation of p53 was detected only in wild‐type p53 harbouring cells. Together, our results suggest that santin and cirsimaritin exhibit promising anti‐cancer activity in cultures of digestive system cancer cells.

## 1 INTRODUCTION

Numerous studies provide evidence for the potential beneficial effect of flavonoids in cancer chemoprevention and inducing cancer cell death.^1^, ^2^ Flavonoids are low molecular weight, secondary plant metabolites characterized by a benzo‐γ‐pyrone core. This broad class of phenolics contains over 4000 compounds.^3^ Due to their wide distribution in the leaves, flowers, fruits, bark and seeds of plants, flavonoids are part of human diet and herbal medicine. Flavonoids occur in plant cells primarily as glycosides and polymers that need to be degraded before absorption.^4^ Hydrolysis of flavonoid glycosides is catalysed by small intestine hydrolases and human intestinal microflora. Absorbed aglycones are metabolized by glucuronidation, sulfation and *O*‐methylation in enterocytes, or transported with subsequent conjugation in the liver. Consequently, orally administered flavonoids reach the highest concentration and thus exert the most potent effect in the digestive tract and liver.

Downy birch (*Betula pubescens* Ehrh.) and silver birch (*Betula pendula* Roth) are deciduous trees characterized by their silver‐white bark. Downy birch commonly grows in Europe and northern Asia, particularly in damp soils, while silver birch prefers less moist soils.^5^ Dried fresh leaves of both birch species are used in Polish traditional medicine as a diuretic.^6^ The major constituents of the leaves that probably cause the diuretic effect are flavonoids, hyperoside and other quercetin glycosides, as well as glycosides of kaempferol and myricetin, which together comprise up to 3% of the mass of birch leaves.^7^, ^8^ Birch buds are also used as a diuretic, mainly in Russian traditional medicine and to a lesser extent in Poland.^9^ The composition and form of flavonoids in buds and leaves are different. In the leaves, flavonoids exist as glycosides while the buds are characterized by high content of aglycones, mainly various methyl ethers of naringenin, apigenin and kaempferol.^10^ In vitro studies show higher cytotoxicity of flavonoid aglycones when compared with glycosides.^11^ In addition indeed, alcohol extracts from silver birch leaves were shown to exert low cytotoxicity in glioma, breast cancer and epidermoid carcinoma cells.^12^ However, flavonoids contained in birch extracts were reported to efficiently induce cancer cell death by various mechanisms. For instance, isoquercitrin promotes autophagy and subsequently activates mitochondrial‐dependent pathway leading to increase in the level of active caspase‐3 in liver cancer.^13^ Mitochondrial pathway of apoptosis induced by hyperoside in colorectal cancer is triggered by p53.^14^ Apigenin suppress cell cycle progression through G2/M arrest and promotes p53‐dependent mitochondrial pathway of apoptosis in gastrointestinal cancers.^15^, ^16^, ^17^ However, decrease in viability of apigenin‐treated liver cancer cells through induction of autophagy was also reported.^18^ Both mitochondrial and extrinsic pathways are involved in induction of apoptosis in colorectal cancer cells treated with kaempferol.^19^ Our recent report has shown that extracts of birch buds exhibit anti‐cancer activity.^9^ Therefore, it is necessary to investigate the effect of birch bud‐derived flavonoid aglycones on cancer cell death.

The aim of this study was to quantify the content of selected flavonoids in birch buds, assess their antitumor effect and elucidate the molecular mechanism of flavonoid‐mediated anti‐cancer activity. The cytotoxic activity was investigated for ten flavonoids previously isolated from birch buds.

## 2 MATERIALS AND METHODS

### 2.1 Quantification of the flavonoid content in birch bud extracts

Carbon dioxide supercritical extraction (SFE) of birch buds was described in our previous publication.^9^ The later optimisation of the procedure presented in our further study provided an increase in the yield of the extract from the buds of *B*. *pubescens* and *B*. *pendula* to 19.7 ± 1.7% and 25.3 ± 1.3%, respectively. All flavonoids studied in this work were isolated from the obtained extracts by column chromatography, identified by mass spectrometry and NMR as described in our recent work.^20^

The content of selected flavonoids in the obtained extracts was determined from the results of gas chromatography‐mass spectrometric (GC‐MS) analysis. For this purpose, the mass spectrometric detector was calibrated by analysing a series of six calibration solutions prepared by the weight‐volumetric method. The concentration range of the calibration solutions of flavonoids, isolated in sufficient quantities for the purposes of the work, was 0.06 to 2 mg/mL.

Before GC‐MS analysis, both birch bud extracts and flavonoid aglycones were submitted to a silylation procedure. The birch bud extracts (20 mg) were diluted with 1 mL of pyridine, and 100 μL of N,O‐bis(trimethylsilyl)‐trifluoroacetamide containing 1% trimethylchlorosilane was added. For each calibration solution (1 mL), 100 μL of silylating agent was added. The obtained mixtures were heated for 30 min at 60°C.

Quantitative analysis of flavonoids in birch bud extracts was conducted using an Agilent 7890A gas chromatograph with an Agilent 5975C mass spectrometer (Agilent, USA). A chromatographic separation was performed on a HP‐5MS capillary column (30 m × 0.25 mm × 0.25 μm) at a helium flow rate of 1 mL/min. The initial column temperature was 50°C, rising to 325°C at3°C/min, and the final temperature was held for 10 min. The injector worked in a split mode (1:10) at a temperature of 300°C. The ion source and quadrupole temperatures were 230°C and 150°C, respectively. Electron ionization mass spectrums (EIMS) were obtained at ionization energy 70 eV.

To ensure selectivity in the separation of birch bud extracts, which are complex multicomponent mixtures, GC‐MS analysis was carried out in the monitoring mode of selected ions (SIM), having the highest intensity in the mass spectrum of each of the compounds being determined. In the case of silanized flavonoids containing a TMS group at the C5 position, the most intense peaks are [M ‒ 15]^+^, which are formed as a result of the loss of the CH_3_ group by the molecular ion.^21^

The detection of the TMS derivatives of 7,4'‐dimethylnaringenin, sakuranetin and santin was carried out using ions at m/z 357, 415 and 473, respectively, while in the mass spectra of kumatakenin, cirsimaritin and ermanin, the ion at m/z 473 has the highest intensity. The GC‐MS analyses were performed in triplicate for both birch bud extracts, as well as for each calibration sample. Table 1 shows the results of the quantitative determination of six flavonoids, as well as the regression equations and values of the determination coefficients *R* ^2^. The mass spectra of all quantified flavonoids, as well as the resulting calibration plots, are provided in Supplemental Information (Figure S1, Figure S2, Table S1).

**TABLE 1:** Quantification of selected flavonoids in *B*. *pubescens* and *B*. *pendula* bud SFE extracts

Compound	Concentration in SFE extract (mg/g)		Calibration equation	*R* ^2^
	*Betula pubescens*	*Betula pendula*		
Santin	17.71 ± 0.85	7.35 ± 0.22	Y = 2E−08x + 0.0883	0.995
Cirsimaritin	4.21 ± 0.16	3.79 ± 0.26	Y = 1E−08x + 0.0641	0.996
Ermanin	4.57 ± 0.28	4.25 ± 0.23	Y = 1E−08x + 0.0641	0.996
Sakuranetin	21.62 ± 1.04	4.08 ± 0.36	Y = 1E−07x + 0.1039	0.998
Kumatakenin	10.92 ± 0.71	6.38 ± 0.56	Y = 1E−08x + 0.0641	0.996
7,4'‐Dimethylnaringenin	62.75 ± 4.76	‐	Y = 1E−8x + 0.0yz	‐
Total	121.78 ± 7.8	25.85 ± 1.63		

### 2.2 Cell culture

Colorectal adenocarcinoma cells (DLD‐1), gastric adenocarcinoma cells (AGS) and normal human skin fibroblasts (CCD25Sk) were purchased from the American Type Culture Collection (Manassas, VA, USA). Hepatocellular carcinoma cells (HepG2) were obtained from Sigma‐Aldrich (USA). Cells were grown in Dulbecco's Modified Eagle Medium (PAN‐Biotech, Germany) supplemented with 10% foetal bovine serum (Gibco, USA) and 1% penicillin/streptomycin (Gibco, USA) in 95% air and 5% carbon dioxide at 37°C.

### 2.3 Cell viability assay

Viability of cells was determined using the MTT assay. Cells were plated in 96‐well plates at 1 × 10^4^ cells per well and allowed to adhere for 24 h. Cells were treated with various concentrations of flavonoids isolated from birch buds and apigenin (SMB00702, Sigma‐Aldrich, USA) used as reference agent (6.25, 12.5, 25, 50 and 100 μM) for 24 or 48 h. MTT (3‐(4,5‐Dimethyl‐2‐thiazolyl)‐2,5‐diphenyl‐2H‐tetrazolium bromide, Sigma‐Aldrich, USA) solution was added to each well, and cells were incubated at 37°C for 4 h. The medium was then removed, and formazan crystals were dissolved in 100 μL of DMSO and 12.5 μL of Sorensen's glycine buffer on a plate shaker. Absorbance was measured at 570 nm using a microplate reader. IC_50_ values were calculated using GraphPad Prism software.

### 2.4 DNA biosynthesis

Cells were plated in 24‐well plates at 4 × 10^4^ cells per well and allowed to adhere for 24 h. Cells were treated with the various concentrations of flavonoids (25, 50 and 100 μM) in 1 mL medium containing 0.5 μCi [^3^H]‐thymidine (#MT6037, Hartmann Analytic, Germany) for 48 h. Medium was discarded, and cells were rinsed three times with phosphate buffered saline (PBS), and solubilized with 1 mL of 0.1 M sodium hydroxide containing 1% sodium dodecyl sulphate. Then, 9 mL of scintillation liquid (PerkinElmer, USA) was added, and radioactivity incorporated into DNA was measured using a scintillation counter.

### 2.5 Colony formation assay

The method description is available as Supplemental Information.

### 2.6 Apoptosis assay

The method description is available as Supplemental Information.

### 2.7 Western immunoblot

The method description is available as Supplemental Information.

### 2.8 Immunofluorescence microscopy

The method description is available as Supplemental Information.

### 2.9 Statistical analysis

The results were analysed in GraphPad Prism software using a one‐way ANOVA followed by Tukey's test, accepting *p* < 0.05 as significant.

## 3 RESULTS

### 3.1 Quantitative content of selected flavonoids in SFE extracts of birch buds

The results of the quantitative analysis of the selected flavonoids in the extracts of both birch species, carried out using GC‐MS, are presented in Table 1. The total flavonoid content in the extract of downy birch (121.78 ± 7.8 mg/g) was 4.7 times higher than in the extract from silver birch buds (25.85 ± 1.63 mg/g). It was found that the content of santin in the downy birch buds (17.71 ± 0.85 mg/g) is over twice as high as in the silver birch buds (7.35 ± 0.22 mg/g). An even greater difference (5.3‐fold) in the content was noted for sakuranetin. Moreover, the principal flavonoid constituent of the downy birch buds was naringenin‐7,4'‐*O*‐dimethyl ether, which was not detected in the extract of silver birch buds. The content of cirsimaritin and ermanin was about 4 mg/g in both extracts.

### 3.2 Flavonoids isolated from birch buds exert differential effects on viability of digestive system cancer cells and fibroblasts

**FIGURE 1:**
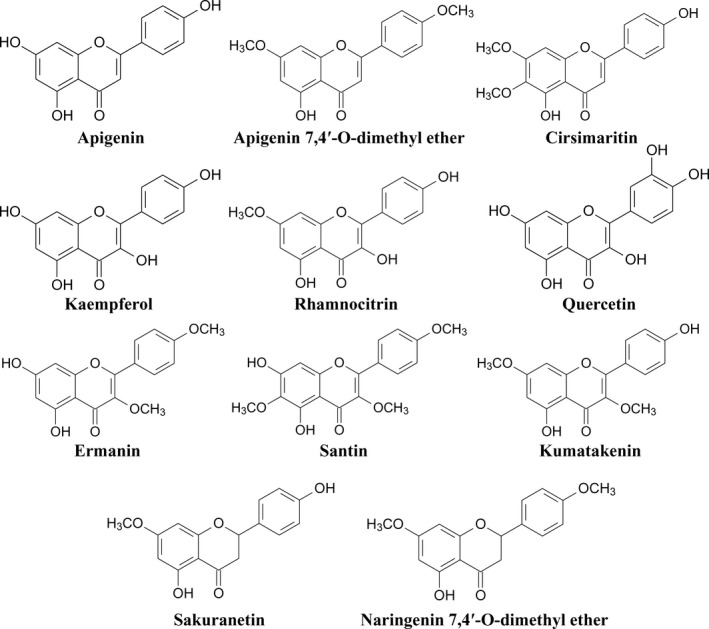
Chemical structures of the ten flavonoids isolated from *Betula pubescens* and *Betula pendula* buds, and apigenin (the reference agent)

The cytotoxic effect of ten flavonoids isolated from birch bud extract: apigenin 7,4'‐*O*‐dimethyl ether, cirsimaritin, ermanin, kaempferole, kumatakenin, naringenin 7,4'‐*O*‐dimethyl ether, quercetin, rhamnocitrin, sakuranetin and santin (Figure 1) on AGS, HepG2 and DLD‐1 cancer cells and normal fibroblasts was evaluated at various time intervals using the MTT assay. Obtained results are provided in Supplemental Information (Figure S3). For the most cytotoxic compounds, half maximal inhibitory concentration (IC_50_) values were determined (Table 2). Flavonoids with IC_50_ values below 50 µM were considered to be active.

**TABLE 2:** IC_50_ values of the flavonoids in gastric adenocarcinoma AGS cells, colorectal adenocarcinoma DLD‐1 cells, hepatocellular carcinoma HepG2 cells and normal human skin fibroblasts (IC_50_ values below 50 μM are shown)

**24 h**				
	IC_50_ values (μM)			
	AGS	DLD−1	HepG2	Fibroblasts
apigenin−7,4'‐*O*‐dimethyl ether				23.6 ± 2.2
**48 h**				
apigenin−7,4'‐*O*‐dimethyl ether				12.9 ± 0.5
cirsimaritin	31.7 ± 2.4			
ermanin	49.3 ± 3.3	49.3 ± 2.5		
santin	17.1 ± 0.8	28.0 ± 1.3	19.8 ± 0.9	
apigenin	18.2 ± 1.3	43.6 ± 2.0		
**72 h**				
apigenin−7,4'‐*O*‐dimethyl ether				19.7 ± 1.3
cirsimaritin	17.1 ± 1.4			
ermanin	44.5 ± 3.1			
kumatakenin				41.6 ± 3.5
quercetin	28.4 ± 2.5			
santin	13.9 ± 1.2	36.5 ± 2.5	22.7 ± 1.6	
apigenin	23.4 ± 1.4		37.9 ± 2.9	

Among the tested compounds, santin was more cytotoxic than apigenin in all cancer cell lines; however, upon 24 h treatment, IC_50_ values for both compounds were higher than 50 μM. After 48 h exposure, IC_50_ values for santin in AGS, DLD‐1 and HepG2 cells were 17.1 ± 0.8, 28.0 ± 1.3 and 19.8 ± 0.9 μM, respectively, whereas for apigenin 18.2 ± 1.3, 43.6 ± 2.0 and >50 μM. IC_50_ values for 72 h treatment with santin were 13.9 ± 1.2, 36.5 ± 2.5 and 22.7 ± 1.6 μM, respectively. These values for apigenin were found to be 23.4 ± 1.4, >50 and 37.9 ± 2.9 μM. Cirsimaritin showed comparable cytotoxicity to apigenin in AGS cells (IC_50_ values for 48 h and 72 h of treatment were 31.7 ± 2.4 and 17.1 ± 1.4 μM, respectively) but it was lower in HepG2 and DLD‐1 cell lines. In contrast, the cell viabilities were slightly decreased or unchanged in cells treated with the other eight flavonoids. Among the less active flavonoids, ermanin showed marked cytotoxicity (reduction in viability by over 80% after 48 and 72 h incubation) in all cancer cells, but only at the highest concentration used in the study (100 μM), and quercetin was moderately active in AGS cells (IC_50_ for 72 h treatment was 28.4 ± 2.5 μM). Importantly, santin, cirsimaritin and apigenin were more active in cancer cells than in fibroblasts, and for these compounds, IC_50_ values were >50 μM in all time periods. In contrast, apigenin 7,4'‐*O*‐dimethyl ether showed marked and selective cytotoxicity in fibroblasts. IC_50_ values for this compound were found to be 23.6 ± 2.2 (24 h), 12.9 ± 0.5 (48 h) and 19.7 ± 1.3 μM (72 h). Cytotoxic activity of all tested flavonoids increased when incubation time was increased from 24 h to 48 h. However, this effect was not consistent when treatment was increased further, from 48 h to 72 h. In this case, the activity of santin and apigenin slightly decreased. Finally, our results demonstrated that DLD‐1 cells are less sensitive to the cytotoxic effects of tested flavonoids than AGS and HepG2 cell lines.

**FIGURE 2:**
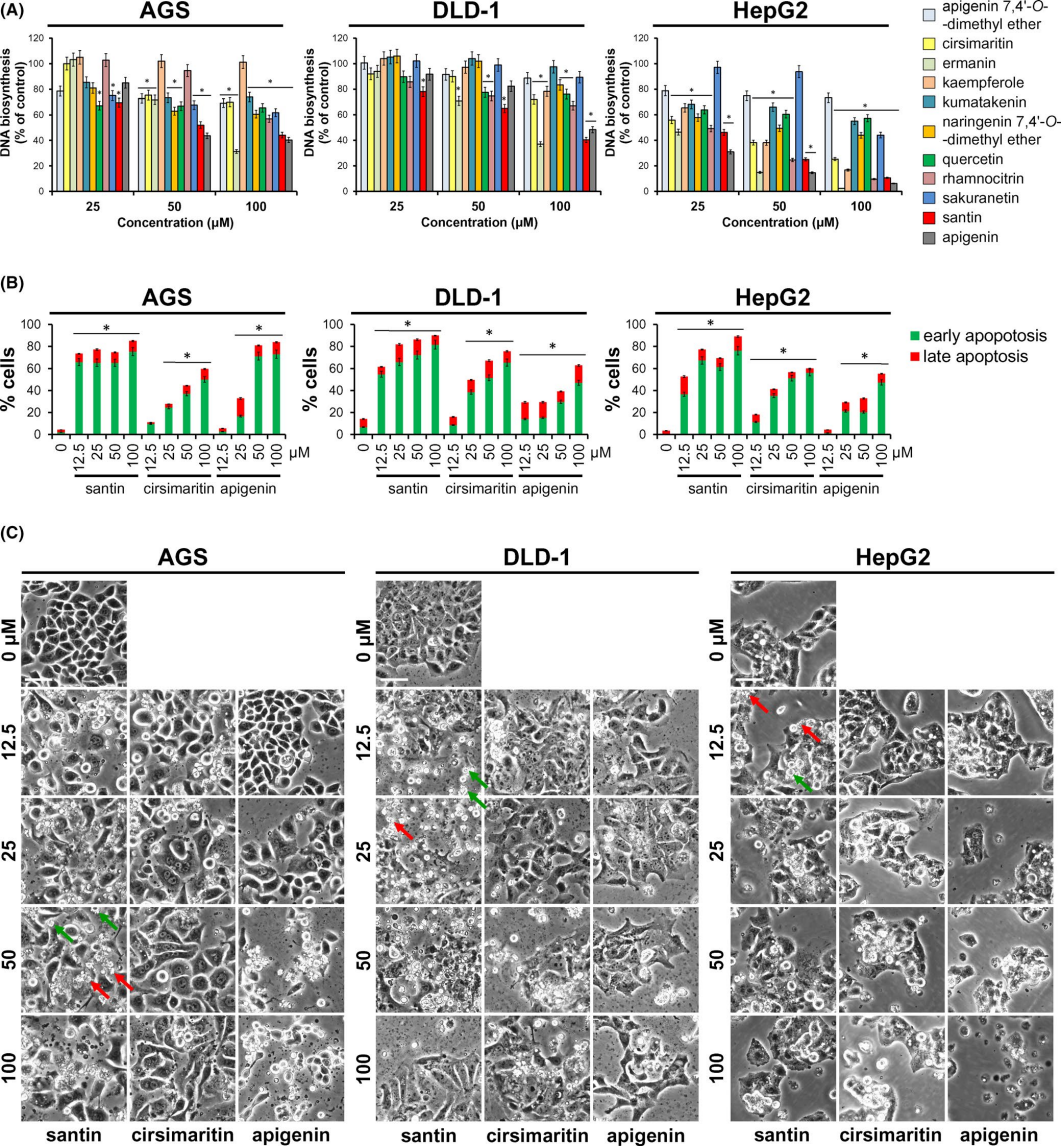
Santin and cirsimaritin decrease viability of cells by induction of apoptosis and inhibition of proliferation. A. Gastric adenocarcinoma AGS cells, colorectal adenocarcinoma DLD‐1 cells, hepatocellular carcinoma HepG2 cells and normal human skin CCD25Sk fibroblasts were treated with different doses of flavonoids for 48 h. DNA synthesis was examined using the [^3^H]‐thymidine incorporation assay. The asterisk indicates *p* < 0.05, when compared to the control group (0.1% DMSO). **B**. AGS, DLD‐1 and HepG2 cancer cells were treated with santin, cirsimaritin and apigenin for 48 h. Cells were stained with annexin V/propidium iodide, and the percentage of apoptotic cells was determined using fluorescence microscopy. Data were showed as mean ±SD for three independent experiments. The asterisk indicates *p* < 0.05, when compared to the control group (0.1% DMSO) **C**. Morphological changes of AGS, DLD‐1 and HepG2 cells after 48 h treatment with santin, cirsimaritin and apigenin. Cell membrane blebbing (green arrows) and formation of apoptotic bodies (red arrows). The length of the scale bar is 50 μm

The observed decrease in cancer cell viability could be the result of impaired cell proliferation rate. Hence, the [^3^H]‐thymidine incorporation assay was performed. Treatment with both santin and apigenin showed significantly decreased [^3^H]‐thymidine incorporation into newly synthesized DNA after 48 h incubation (Figure 2A). Incorporation of [^3^H]‐thymidine in AGS, DLD‐1 and HepG2 cells treated with 50 μM of santin was reduced by 48.0%, 35.0% and 74.9%, respectively. The same concentration of apigenin led to decrease in [^3^H]‐thymidine incorporation by 56.2%, 17.7% and 85.4%. In addition, reduction in [^3^H]‐thymidine incorporation by ermanin (50 μM) was higher in all cell lines (28.2%, 29.1% and 85.2%) when compared with cirsimaritin (24.5%, 10.0% and 53.6%). Similarly to results obtained in the MTT assay, DNA biosynthesis was more affected in AGS and HepG2 cells than in the DLD‐1 cell line. Our data suggest that growth suppression may play a role in the decrease in cell viability induced by santin and cirsimaritin.

### 3.3 Santin and cirsimaritin induce apoptosis of digestive system cancer cells

The above findings indicate that treatment of cancer cells with santin and cirsimaritin results in a significant decrease in cell viability and proliferation. To further explore the mechanism by which these flavonoids decrease cell viability, an annexin V/propidium iodide staining assay was used, followed by fluorescence microscopy. Treatment of cells with santin and cirsimaritin for 48 h resulted in a significant increase in the percentage of apoptotic cells exhibiting green annexin V staining (Figure 2B). Representative images of stained cells are provided in Supplemental Information (Figure S4). The apoptosis‐inducing activity of santin was higher than the activity of cirsimaritin in all tested cancer cell lines. Treatment with 12.5 μM of santin increased the percentage of cells in the early and late stage of apoptosis to 65.8% and 7.6% (AGS), 54.8% and 6.7% (DLD‐1), and 36.6% and 16.1% (HepG2), respectively. The comparable values was achieved by treatment of cells with 50 μM of cirsimaritin: 37.0% and 7.4% (AGS), 51.5% and 15.5% (DLD‐1), and 51.2% and 5.4% (HepG2). Moreover, apoptosis‐inducing activity of santin was higher than the activity of apigenin in all tested cell lines. Following treatment with 50 μM of apigenin, the percentage of early and late apoptotic cells was 71.2% and 9.6% (AGS), 29.7% and 9.4% (DLD‐1), and 20.5% and 12.3%, respectively. The apoptosis‐inducing activity of cirsimaritin was higher in DLD‐1 and HepG2 cells, when compared with apigenin.

Bright‐field microscopic images of cells confirmed apoptotic features in cells treated with flavonoids (Figure 2C). Cells lost their original shape, became round and detached from the plate surface. Cell membrane blebbing and formation of apoptotic bodies were also observed. However, some cells treated with cirsimaritin and santin, even at the highest concentration used in the study, were still adhered to the plate surface. These cells were substantially larger and showed binucleation or multinucleation, for instance, AGS cells treated with cirsimaritin. In contrast, upon apigenin treatment, all or almost all AGS and HepG2 cells were detached from the plate surface.

**FIGURE 3:**
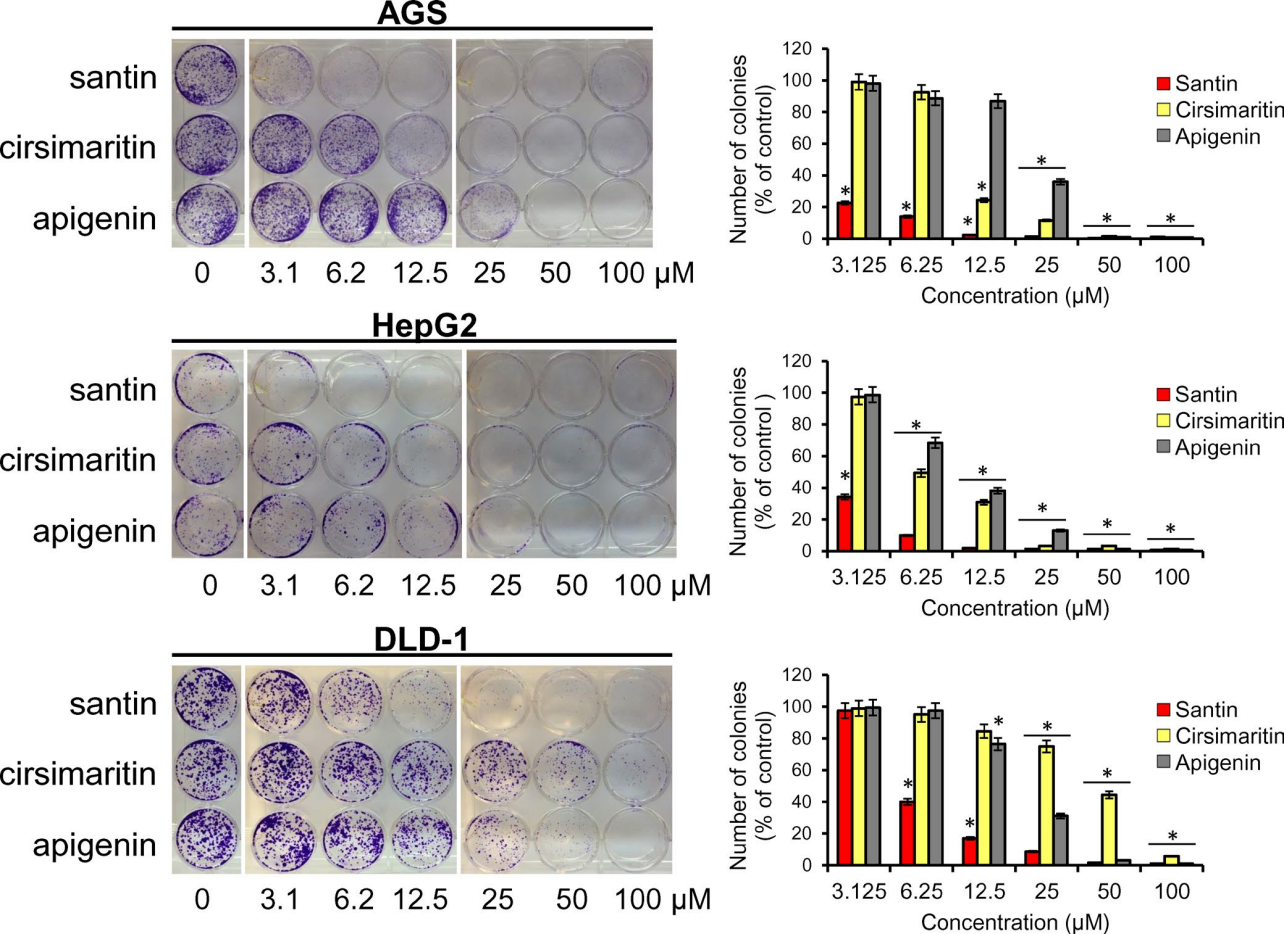
Santin and cirsimaritin treatment lead to impairment in proliferation of surviving cells. Colony formation assay of AGS, DLD‐1 and HepG2 cancer cells treated with different concentrations of santin, cirsimaritin and apigenin for 48 h and then cultured for 7 days. The histogram shows the ratio of clone formation. The control group was normalized to 100%. The asterisk indicates *p* < 0.05 when compared to the 0.1% DMSO‐treated group

In order to determine whether cells treated with santin and cirsimaritin are able to proliferate, a colony formation assay was performed. Exposure of cells to santin resulted in a significant decrease in the number of cell colonies (Figure 3). The lowest concentration of santin (3.125 μM) led to decrease in colony numbers by 77.4% (AGS) and 65.8% (HepG2). DLD‐1 cells showed slightly higher resistance to santin; however, 6.25 μM of santin induced 60% decrease in number of colonies. Santin exhibited a more potent ability to decrease colony formation than apigenin and cirsimaritin in all cell lines. Treatment with 12.5 μM of apigenin led to decrease in number of colonies by 13.2% (AGS), 61.8% (HepG2) and 23.5% (DLD‐1). In contrast, cirsimaritin was more active than apigenin only in AGS cells (12.5 μM decreased number of colonies by 75.7%). Obtained results suggest that cells which survived treatment with santin had impaired ability to proliferate.

These data show that santin and cirsimaritin act as apoptosis inducers and santin has a prolonged proliferation‐suppressing effect.

### 3.4 Santin and cirsimaritin activate intrinsic and extrinsic pathways of apoptosis in digestive system cancer cells

**FIGURE 4:**
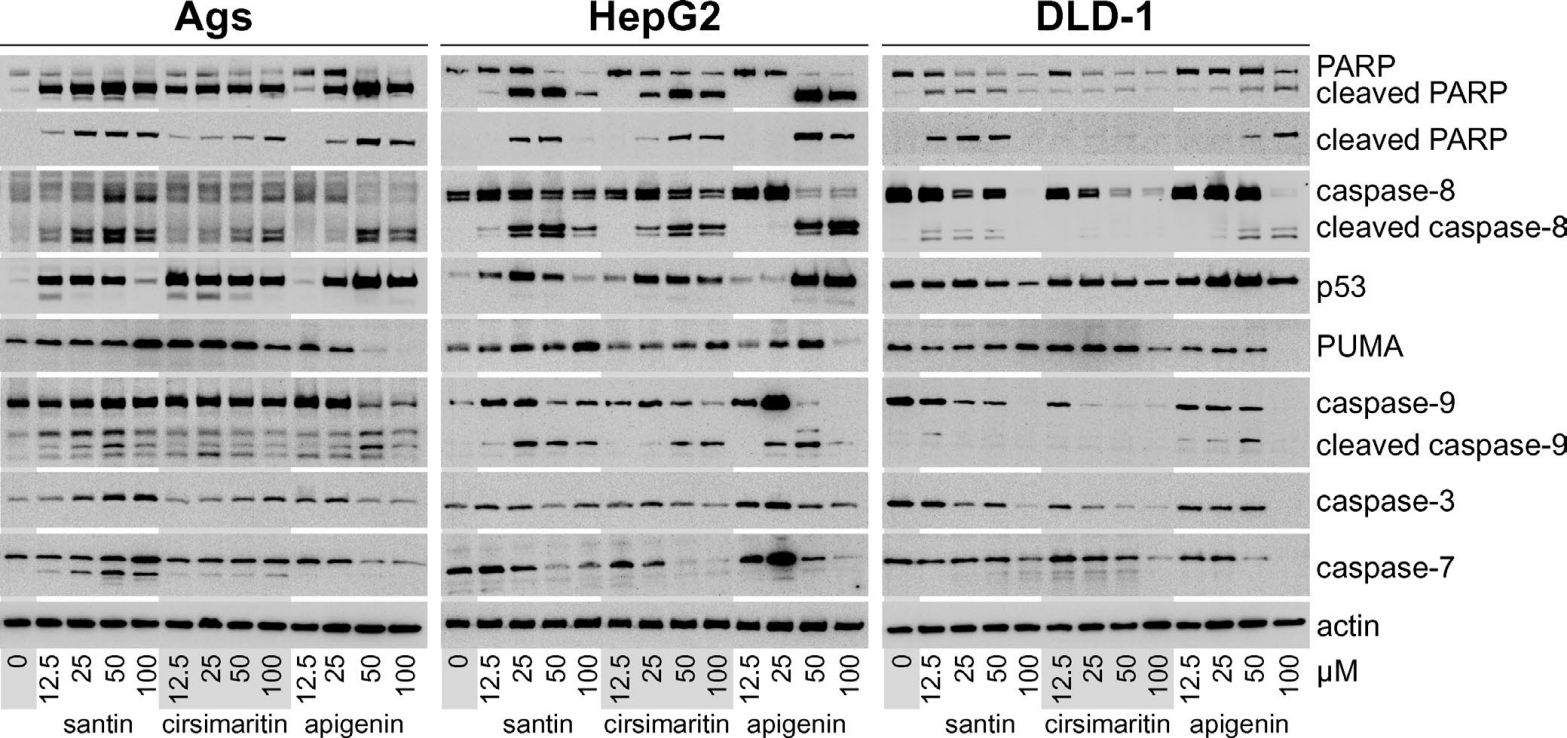
Santin and cirsimaritin activate intrinsic and extrinsic pathways of apoptosis. Western blot analysis for PARP, caspase‐8, p53, PUMA, caspase‐9, caspase‐3 and caspase‐7 in AGS, DLD‐1 and HepG2 cancer cells treated with different concentrations of santin, cirsimaritin and apigenin for 48 h. Actin served as a control for protein loading

**FIGURE 5:**
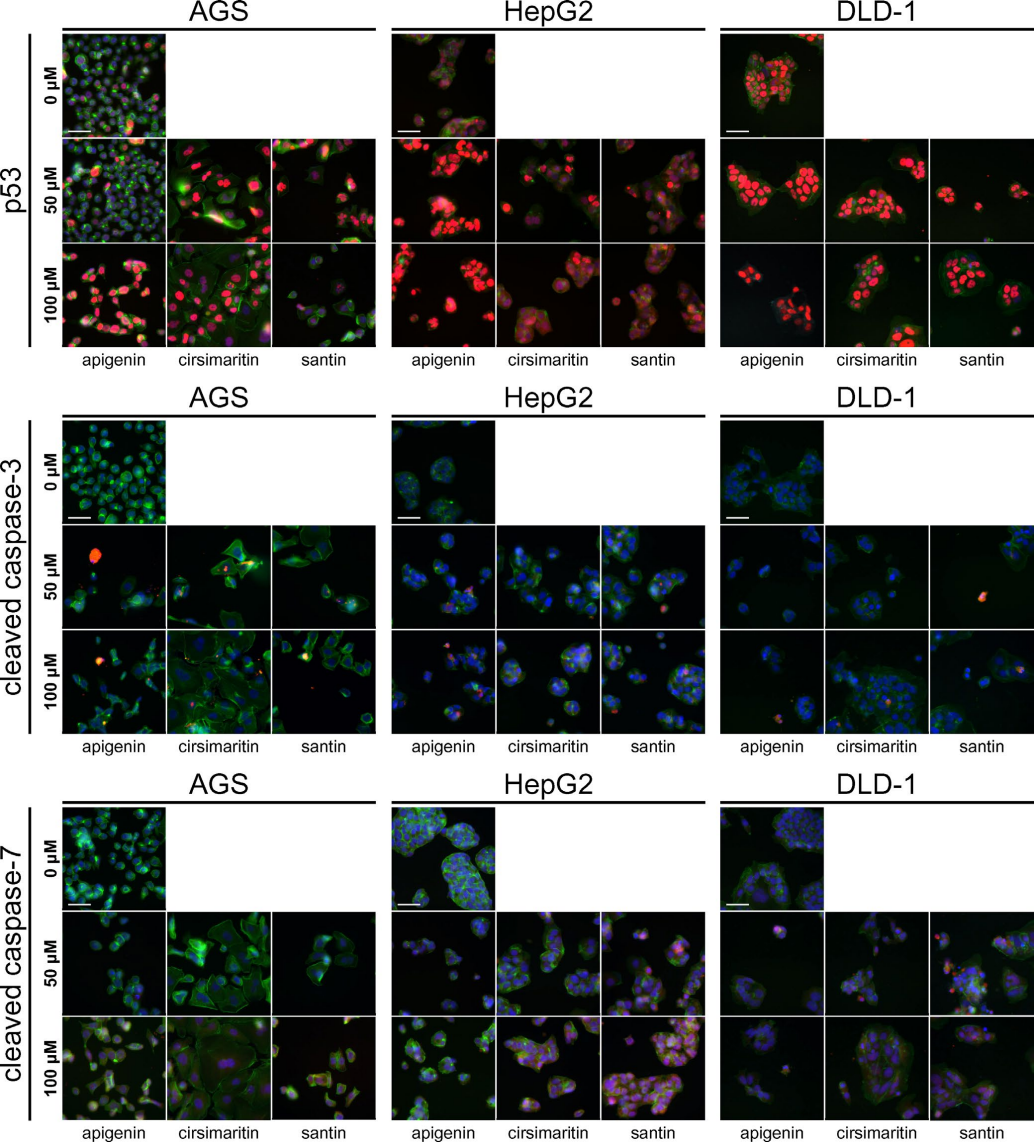
Santin and cirsimaritin induce nuclear accumulation of p53 and activate effector caspases. Immunofluorescence analysis for p53, cleaved caspase‐3 and cleaved caspase‐7 in AGS, DLD‐1 and HepG2 cancer cells treated with different concentrations of santin, cirsimaritin and apigenin for 48 h

To explore the mechanism by which santin and cirsimaritin induce cell apoptosis, the effects of these compounds on the expression of selected pro‐apoptotic proteins were examined using Western blot analysis and fluorescence microscopy. Activation of initiator and effector caspases is a relevant criterion for determination of apoptosis. Both santin and cirsimaritin activate initiator caspase‐8 and initiator caspase‐9, as judged by the increase in levels of cleaved fragments and decrease in levels of pro‐forms (Figure 4). In parallel, the expression of effector caspases, both caspase‐3 and caspase‐7 pro‐forms, is down‐regulated. Analysis of cleaved effector caspases using immunofluorescence showed an increase in signal intensity in cells treated with all tested flavonoids (Figure 5). Consistent with these findings, treating cells with the same concentrations of flavonoids resulted in a decrease in the level of (poly(ADP‐ribose) polymerase) PARP and an increase in the level of cleaved PARP.

Comparison of the impact of the different flavonoids on caspase expression indicates that santin had the most pronounced effect. Activation of initiator caspases in apigenin‐treated cells was generally found at higher concentrations when compared with santin and cirsimaritin. Notably, the expression level of cleaved initiator caspases in DLD‐1 cells treated with cirsimaritin was very low, despite an observed decrease in expression of the pro‐form. Activation of effector caspases was more pronounced in santin‐ and cirsimaritin‐treated cells than in cells treated with apigenin. Likewise, cleavage of PARP and expression of the cleaved form of PARP was low in cirsimaritin‐treated DLD‐1 cells, despite the significant decrease in active PARP. It was also found that santin and cirsimaritin up‐regulated some pro‐forms of caspases, particularly in AGS cells. This phenomenon may indicate uneven activation of transcription factors specific for given cell line.

To get further insight into the potential mechanism by which santin and cirsimaritin promote apoptosis, expression of p53 was determined by Western blot (Figure 4) and fluorescence microscopy (Figure 5). We found that the p53 level increased significantly in santin‐ and cirsimaritin‐treated AGS and HepG2 cells, while it was decreased in DLD‐1 cells. Meanwhile, p53 was up‐regulated in all cell lines treated with apigenin, but a higher concentration of apigenin is needed to induce p53 expression level change compared to santin and cirsimaritin. Consequently, p53 may participate in flavonoid‐induced apoptosis in AGS and HepG2 cells. This hypothesis is reinforced by the increased expression of pro‐apoptotic protein PUMA (p53 up‐regulated modulator of apoptosis) detected by Western blot. Therefore, p53 upregulation may be responsible for the high pro‐apoptotic activity of flavonoids in AGS and HepG2 cells but not in the DLD‐1 cell line.

Taken together, the above results suggest that santin and cirsimaritin promote apoptosis of digestive system cancer cells by induction of intrinsic and extrinsic apoptotic pathways. Additionally, upregulation of p53 by santin and cirsimaritin may contribute to the induction of apoptosis.

## 4 DISCUSSION

Parts of plants from the genus *Betula*, for instance, the leaves, bark, buds and flowers, have been widely used for centuries in traditional medicine.^5^ In addition, *Betula* bark is also a source of triterpenoids, mainly betulinic acid and betulin, which have been extensively studied, both for anti‐cancer properties and for obtaining derivatives.^22^ Results of the present study demonstrated that the santin and cirsimaritin contained in the buds of both investigated birch species exhibit anti‐cancer activity in gastric, colon and liver cell lines. Moreover, the toxic effect of these flavonoids in normal fibroblasts was less pronounced when compared with cancer cells and these results are consistent with report showing higher sensitivity of cancer cells to apoptosis induced by cirsimaritin.^23^ This phenomenon could have clinical implications, as it suggests higher specificity towards cancer cells over normal cells. However, more detailed studies of normal epithelial cells from the digestive tract and hepatocytes are required to support this hypothesis.

Flavonoids are a large group of polyphenolic compounds sharing a common phenylbenzopyrone structure (C6‐C3‐C6). They are classified according to the level of saturation and substitution of the central pyran ring, mainly into flavans, flavones, flavanols, isoflavones, flavonols, flavanones and flavanonols.^24^ The mechanism of anti‐cancer action of flavones and flavonols is associated with cell cycle arrest, disturbance in pro‐survival signalling, apoptosis induction, inhibition of angiogenesis, and antioxidative/pro‐oxidative activity.^2^, ^25^ In our study, we observed a strong apoptosis‐inducing activity of santin and cirsimaritin.

Santin belongs to the flavonols class and was reported to exhibit anti‐inflammatory properties due to the suppression of both cyclooxygenase‐2 (COX‐2) and inducible nitric oxide synthase (iNOS).^26^ This effect of santin treatment might be of great importance, since COX‐2 activity positively correlates with the occurrence and progression of cancer.^27^, ^28^ Presumably, the anti‐inflammatory activity of santin is connected to its ability to attenuate the activity of the transcription factor NF‐κB.^29^ This protein is known to stimulate transcription of the genes encoding COX‐2 and iNOS.^30^ Importantly, NF‐κB is also able to inhibit apoptosis.^31^, ^32^ However, there is no literature indicating the anti‐cancer activity of santin, besides a description showing low activity of this compound as mitotic blocker.^33^ Our results revealed that the viability of cancer cells is affected more strongly by santin than by any other flavonoid isolated from birch buds. Cytotoxic effect of santin was also stronger than in cells treated with apigenin which beneficial activity on digestive system cancer cells was proven in many reports.^15^, ^16^, ^17^, ^18^ In contrast, cirsimaritin belongs to the flavones class, and several studies have shown that cirsimaritin induces the death of various cancer cells, including colon cancer^34^, ^35^ and gastric cancer.^35^ Interestingly, like santin, cirsimaritin inhibits the NF‐κB pathway and down‐regulates COX‐2 and iNOS^36^, ^37^; however, its role in cancer needs to be elucidated. Our study confirms the significant anti‐cancer activity of cirsimaritin. In addition, the obtained results reveal the mechanism of cell death induced by cirsimaritin and santin.

Our study demonstrated that apoptosis is primarily responsible for flavonoid‐induced cell death. In the liver and digestive tract, the apoptotic process triggered by flavones and flavonols is associated with increased or decreased reactive oxygen species (ROS) production,^19^, ^38^, ^39^ increase in p53 expression,^19^ decrease in the mitochondrial membrane potential,^39^, ^40^ elevated Bax/Bcl‐2 ratio,^18^, ^39^ release of cytochrome c from the mitochondria to the cytosol,^38^ activation of caspase‐9,^18^, ^19^, ^38^, ^39^ activation of caspase‐8^39^ and activation of caspase‐3.^18^, ^19^, ^38^, ^39^, ^41^ Consistent with these data, our study has demonstrated that santin and cirsimaritin trigger the intrinsic pathway of apoptosis (activation of caspase‐9). These results are consistent with the finding that cirsimaritin activates caspase‐9 and caspase‐3.^23^ Moreover, the detected induction of the extrinsic pathway of apoptosis (activation of caspase‐8) by both flavonoids may reinforce the apoptosis‐triggering signal. Substrates for caspases‐catalysed proteolysis, such as gelsolin, α‐fodrin, ICAD (inhibitor of caspase‐activated deoxyribonuclease), pRb (retinoblastoma tumour suppressor protein), topoisomerase I, PKCs (protein kinases C), lamins and PARP are found both, in cytoplasm and nucleus.^42^, ^43^ Immunofluorescence staining showed expression of cleaved forms of executioner caspases in cytoplasm and nucleus making them close to the substrates. It is worth mentioning that santin and cirsimaritin up‐regulated some pro‐forms of caspases, particularly in AGS cells. This phenomenon may occur due to increased transcription of caspase genes or increased mRNA stability during flavonoid‐induced apoptosis in certain cell lines.^44^, ^45^, ^46^

Up‐regulation of p53 in wild‐type p53‐containing AGS and HepG2 cells suggests a partial contribution of p53 to the promotion of apoptosis. P53 is a well‐characterized tumour suppressor transcription factor that gets activated by cellular stress such as DNA damage, oxidative stress, oncogene activation, hypoxia and telomere shortening.^40^ Importantly, p53 has been reported to play a pivotal role in flavone‐ and flavonol‐induced apoptosis^47^, ^48^; however, p53‐independent cell death was also described.^49^, ^50^ Indeed, our results also indicate significant toxicity of santin and cirsimaritin in both cancer cells expressing wild‐type *TP53* (AGS, HepG2) and colon adenocarcinoma cells harbouring a *TP53* mutation (DLD‐1).^51^ Moreover, increase in expression of PUMA, one of the key proteins for which gene transcription is up‐regulated by p53 confirm increased transcriptional activity of p53 in AGS and HepG2 cells.^52^

The other flavonoids from birch buds showed moderate or weak cell death‐inducing activity. Both flavanones (sakuranetin and naringenin 7,4'‐*O*‐dimethyl ether) carry a methoxyl group at the C7 position. The lack of a C2‐C3 double bond, which is a hallmark of the flavanones class, leads to loss of the planar structure of rings in the benzo‐γ‐pyrone molecule, which, in turn, is associated with a decrease in toxicity.^53^ Consistent with this, both isolated flavanones showed the lowest toxicity in our study. The flavones class has a saturated double bond (C2=C3). Similarly to the flavanones, both flavones (apigenin 7,4'‐*O*‐dimethyl ether and cirsimaritin) carry a methoxyl group at C7 and showed lower toxicity than apigenin. Research on the impact of the methoxyl group at C7 in flavones on cell death provides conflicting results depending on the cell line.^54^, ^55^ Cirsimaritin, which contains a free hydroxyl group at C4’ and an additional methoxyl group at C6, exhibits strong apoptosis‐inducing activity. It has been shown that the introduction of a methoxyl group to flavonoids increases their lipophilicity and membrane permeability, leading to the accumulation of molecules in the cell.^56^ Flavonols differ from flavones, with a hydroxyl group at C3 that is associated with reduced cytotoxicity.^53^, ^57^ Consistent with these findings, three flavonols with a free hydroxyl group at C3 (kaempferol, quercetin and rhamnocitrin) showed less pronounced toxicity than apigenin. However, three flavonols with a methylated hydroxyl group at C3 (ermanin, santin and rhamnocitrin) showed a differential effect on cell viability. Kumatakenin, which has a methylated hydroxyl group at C7, exhibits low toxicity, whereas free hydroxyl group‐containing ermanin and santin significantly decreased cancer cell viability. This difference in activity may also be related to the methylation of the hydroxyl group at C4’, since this modification was shown to have a different effect depending on cell type.^53^, ^55^, ^58^ To conclude, the negative influence of the hydroxyl group at C3 is, at least in part, removed by methylation. Additionally, like cirsimaritin, santin is C6‐methoxylated. Flavonoids exhibiting this type of structure, such as 3,6‐dimethoxyapigenin, were found to have significant antiproliferative activity in a variety of cancer cell lines.^59^ Therefore, it can be concluded that the methoxyl group at C6 probably leads to an increase in the cytotoxicity of flavonoids; however, this finding should be supported by further research.

It has been previously described that triterpene *seco*‐acids isolated from the SFE extract of *Betula pubescens* buds induce apoptosis in gastric and colon cancer cells.^60^ Our present study indicates that flavonoids, at least in part, may contribute to the previously reported cytotoxic activity of the SFE extract.^9^

In conclusion, our studies indicate that santin and cirsimaritin induce apoptotic cell death via the intrinsic and extrinsic pathways of apoptosis in cancer cells. Further investigation using in vivo assays such as tumour xenografts to test santin and cirsimaritin efficacy would be a natural extension to our current study.

## CONFLICT OF INTEREST

The authors confirm that there are no conflicts of interest.

## AUTHOR CONTRIBUTION

**Lukasz Szoka:** Funding acquisition (supporting); Investigation (lead); Methodology (lead); Visualization (lead); Writing‐original draft (lead). **Jolanta Nazaruk:** Investigation (supporting). **Marcin Stocki:** Investigation (supporting); Visualization (supporting). **Valery Isidorov:** Conceptualization (lead); Funding acquisition (lead); Writing‐review & editing (lead).

## Supporting information

Supplementary Material

## Data Availability

The data that support the findings of this study are available from the corresponding author upon reasonable request.
