# Supplementary material for: Santin and cirsimaritin from Betula pubescens and Betula pendula buds induce apoptosis in human digestive system cancer cells
Source: J Cell Mol Med. 2021 Nov 9;25(24):11085–96. doi: 10.1111/jcmm.17031 (PMC8650031; doi:10.1111/jcmm.17031)
Supplement: Supplementary file 1 — Supplementary Material [file JCMM-25-11085-s001.pdf]

Supplementary materials associated with Łukasz Szoka, Jolanta Nazaruk, Marcin Stocki and Valery Isidorov article **Cytotoxicity of flavonoids from *Betula pubescens* and *Betula pendula* buds in human digestive system cancer cells**

Corresponding author: lukasz.szoka@umb.edu.pl

This document contains the following Supplementary Materials:

Materials and methods:
2.5. Colony formation assay
2.6. Apoptosis assay
2.7. Western immunoblot
2.8. Immunofluorescence microscopy

Figure S1. Calibration plots obtained as a result of GC-MS analysis of artificial solutions of flavonoids in the selective ion monitoring (SIM) mode (m/z = 357, 415, 443, 473).

Figure S2. Mass spectra of flavonoids quantified in extracts from birch buds.

Figure S3. Flavonoids from birch buds differentially affect viability of digestive system cancer cells and fibroblasts. Gastric adenocarcinoma AGS cells, colorectal adenocarcinoma DLD-1 cells, hepatocellular carcinoma HepG2 cells and normal human skin CCD25Sk fibroblasts were treated with different doses of flavonoids for 24, 48 and 72 h. Cell viability was determined using the MTT assay. 0.1% DMSO was used as a vehicle control. Data were expressed as mean ± SD (n = 3). The asterisk indicates $P < 0.05$.

Figure S4. Representative merged photographs of cells stained with annexin V-FITC conjugate (green fluorescence), propidium iodide (red fluorescence), and Hoechst 33342 (blue fluorescence), visualised by fluorescence microscopy. Scale bar = 50 µm.

Table S1. Ions used for quantification of individual flavonoids.

and

Full-length blots

2.5. Colony formation assay

In total, 250 cells were plated in each well of 12-well plates and allowed to adhere for 24 h. Cells were treated with various concentrations of flavonoids (3.125, 6.25, 12.5, 25, 50 and 100 μM) for 48 h. Medium was discarded and fresh medium was added. After seven days, culture medium was removed and cells were washed once with PBS. Then, cells were fixed with 4% formaldehyde and stained with 0.1% crystal violet.

2.6. Apoptosis assay

Cells were seeded at a density of 1×10^5 cells per well in 6-well plates and allowed to adhere for 24 h. Cells were treated with various concentrations of flavonoids (12.5, 25, 50 and 100 μM) for 48 h. Floating and adherent cells were collected and assayed using a Dead Cell Apoptosis Kit with annexin V-fluorescein isothiocyanate (FITC) and propidium iodide (PI), for flow cytometry (#V13242, Thermo Fisher Scientific, USA), according to the manufacturer's protocol. Briefly, cells were dispersed in 100 μL annexin-binding buffer containing 5 μL annexin V-FITC conjugate solution, 1 μg/mL PI and 1 μg/mL Hoechst 33342, and incubated for 15 min at room temperature. Then 400 μL of annexin-binding buffer was added, and the cell suspension was transferred to 96-well plates and visualised using fluorescence microscopy on a BD Pathway 855 system (Becton Dickinson, USA). Early apoptotic cells showed green and blue fluorescence, while late apoptotic cells showed green, red and blue fluorescence.

2.7. Western immunoblot

Adherent and floating cells were harvested and sonicated. Obtained homogenates were supplemented with 1% protease and phosphatase inhibitor cocktail (#78440, Thermo Fisher Scientific, USA). A Lowry assay was performed to quantify protein content in the homogenates. Proteins (20–40 μg) were resolved on 7.5%, 10% or 12% SDS-PAGE gels using the Mini-Protean Tetra system (Bio-Rad, USA). Proteins were transferred to nitrocellulose membranes (Bio-Rad, USA) using the Mini Trans-Blot Cell wet blotting system (Bio-Rad, USA). Membranes were blocked with 5% skim milk for 1 hour at room temperature and probed overnight at 4 °C with primary antibodies. The following antibodies were used: anti-caspase-9 antibody (#9508, 1:1000), anti-caspase-8 antibody (#9746, 1:1000), anti-caspase-3 antibody (#9662, 1:1000), anti-caspase-7 (#12827, 1:1000), anti-PUMA (#12450, 1:1000), anti-PARP (#9542, 1:1000) and anti-cleaved PARP antibody (#5625, 1:1000), purchased from Cell Signaling Technology (USA); anti-p53 antibody (#sc-126,

1:500) obtained from Santa Cruz Biotechnology (USA); and anti-actin antibody (#A2066, 1:2000) purchased from Sigma-Aldrich (USA). After extensive washes, a secondary antibody solution in 5% skim milk (anti-mouse IgG-HRP, Sigma-Aldrich, #A9044, 1:5000; anti-rabbit IgG-HRP, Sigma-Aldrich, #A9169, 1:5000) was added for 1 h at room temperature. Membranes were incubated with ECL-HRP substrate (GE Healthcare, USA) and the signal was detected using the BioSpectrum Imaging System (Ultra-Violet Products, Ltd., UK).

2.8. Immunofluorescence microscopy

Cells were plated in 96-well plates at 1×10^4 cells per well and allowed to adhere for 24 h. Cells were treated with the respective concentration of flavonoids and incubated for 48 h. Medium was removed and cells were fixed at room temperature for 15 minutes with 4% paraformaldehyde, rinsed three times with PBS, and permeabilised at room temperature for 5 minutes with 0.1% Triton X-100. Then, non-specific binding was blocked with 10% heat-inactivated goat serum in PBS for 1 h at room temperature. The cells were incubated with the following antibodies at 4 °C, overnight: anti-p53 antibody (#sc-126, 1:200) obtained from Santa Cruz Biotechnology (USA), and anti-cleaved-caspase-3 antibody (#9664, 1:200) and anti-cleaved-caspase-7 antibody (#8438, 1:200) purchased from Cell Signaling Technology (USA). After three washes with PBS, cells were incubated with Alexa Fluor 594 conjugated antibody (#A11032, #A11037; Invitrogen, USA) for 1 h in the dark. Cell nuclei were stained with Hoechst 33342 (Invitrogen, USA) and F-actin was stained with Phalloidin-Atto 488 (Sigma-Aldrich, USA). Fluorescent signals were examined and captured using a BD Pathway 855 confocal microscope.

Quantitative determination of flavonoids in SFE extracts of buds of two birch species was carried out by GC-MS method. The mass spectrometric detector was calibrated by analyzing flavonoid solutions in the concentration range 0.06–2.0 mg/ml in the mode of selective monitoring of the most intense ions ($M^+ - CH_3$) in the spectra of TMS derivatives of the determined components (Table S1).

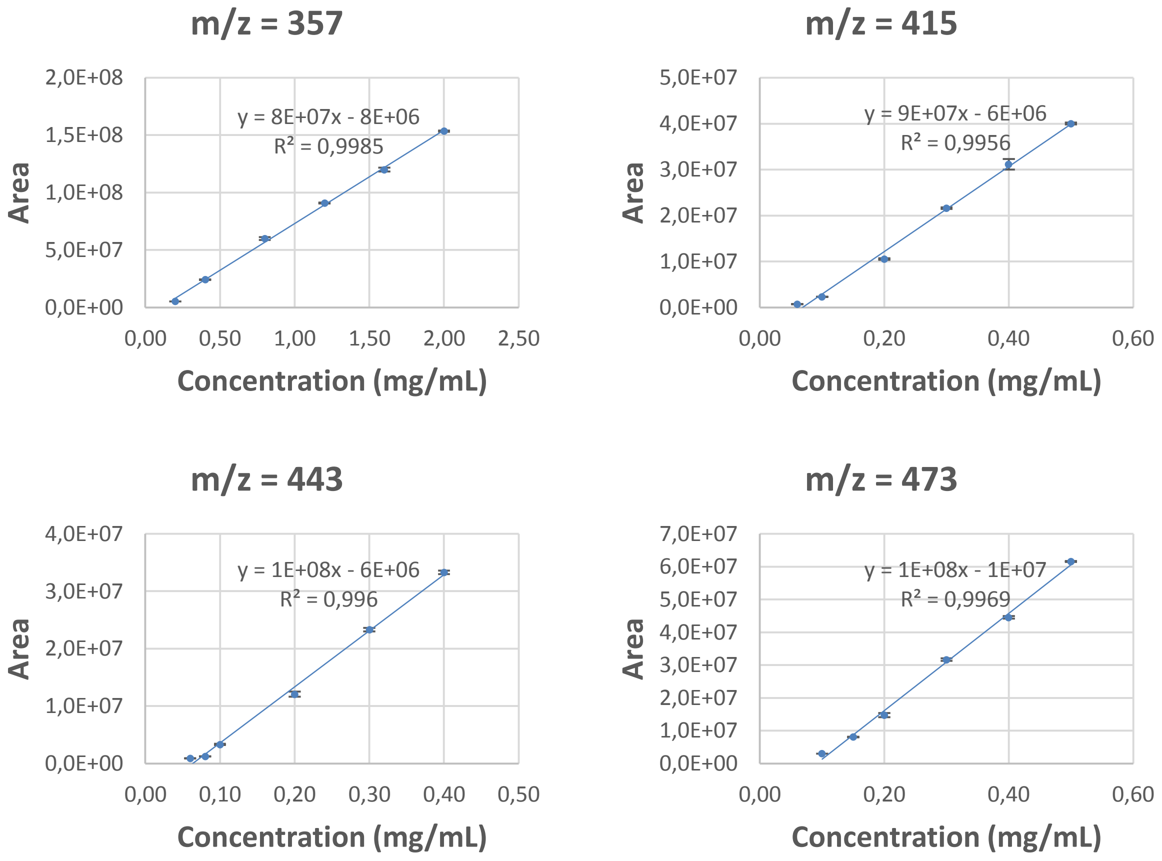

Figure S1. Calibration plots obtained as a result of GC-MS analysis of artificial solutions of flavonoids in the selective ion monitoring (SIM) mode (m/z = 357, 415, 443, 473).

1)

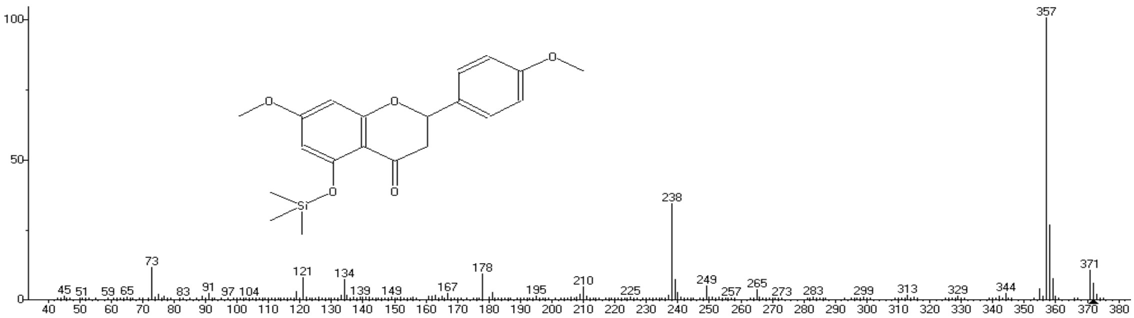
100
50
0
357
238
371
73
45 51 59 65
83 91 97 104
121 134 139 149
167 178 195 210 225
249 257 265 273 283 299 313 329 344
40 50 60 70 80 90 100 110 120 130 140 150 160 170 180 190 200 210 220 230 240 250 260 270 280 290 300 310 320 330 340 350 360 370 380
O
Si

2)

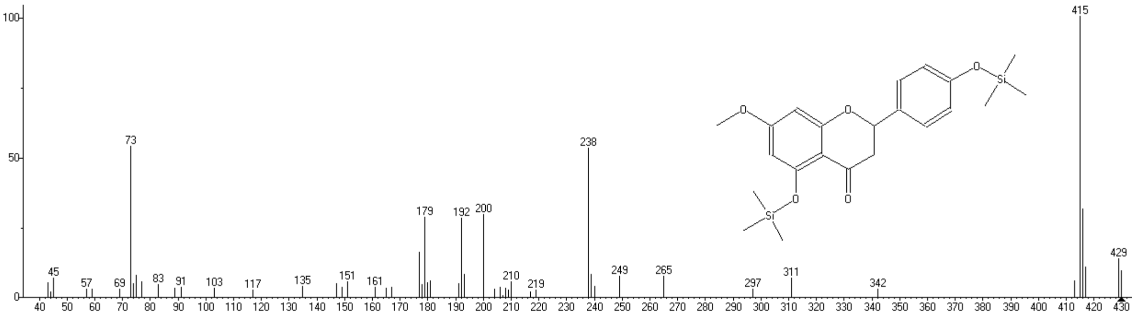
100
50
0
415
73
238
179 192 200
429
45 57 69 83 91 103 117 135 151 161
210 219 249 265 297 311 342
40 50 60 70 80 90 100 110 120 130 140 150 160 170 180 190 200 210 220 230 240 250 260 270 280 290 300 310 320 330 340 350 360 370 380 390 400 410 420 430
O
Si

3)

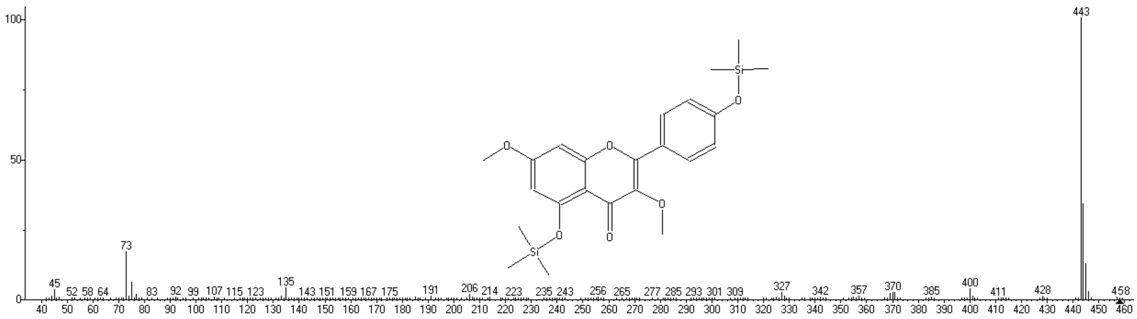
100
50
0
443
73
45 52 58 64 83 92 99 107 115 123 135 143 151 159 167 175 191 206 214 223 235 243 256 265 277 285 293 301 309 327 342 357 370 385 400 411 428 458
40 50 60 70 80 90 100 110 120 130 140 150 160 170 180 190 200 210 220 230 240 250 260 270 280 290 300 310 320 330 340 350 360 370 380 390 400 410 420 430 440 450 460
O
Si

4)

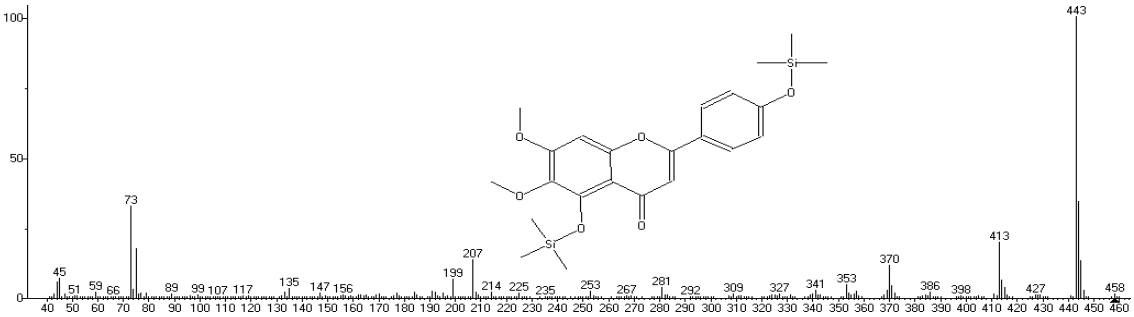
100
50
0
443
73
413
207
370
45 51 59 66 89 99 107 117 135 147 156 199 214 225 235 253 267 281 292 309 327 341 353 386 398 427 458
40 50 60 70 80 90 100 110 120 130 140 150 160 170 180 190 200 210 220 230 240 250 260 270 280 290 300 310 320 330 340 350 360 370 380 390 400 410 420 430 440 450 460
O
Si

5)

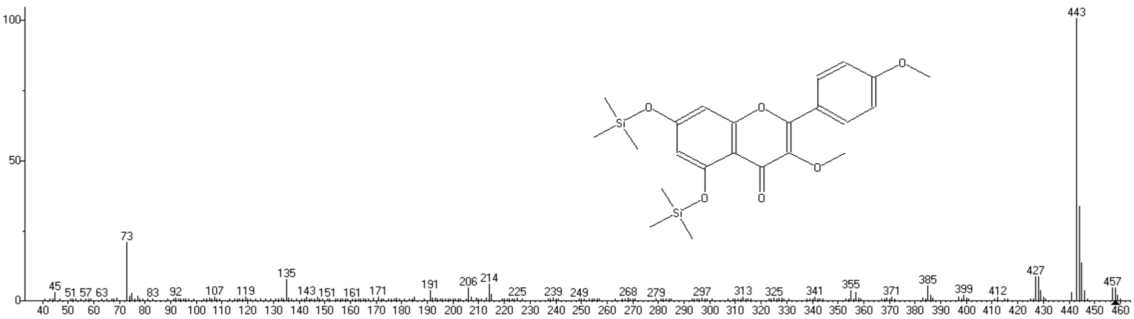
100
50
0
443
73
45 51 57 63 83 92 107 119 135 143 151 161 171 191 206 214 225 239 249 268 279 297 313 325 341 355 371 385 399 412 427 457
40 50 60 70 80 90 100 110 120 130 140 150 160 170 180 190 200 210 220 230 240 250 260 270 280 290 300 310 320 330 340 350 360 370 380 390 400 410 420 430 440 450 460
O
Si

6)

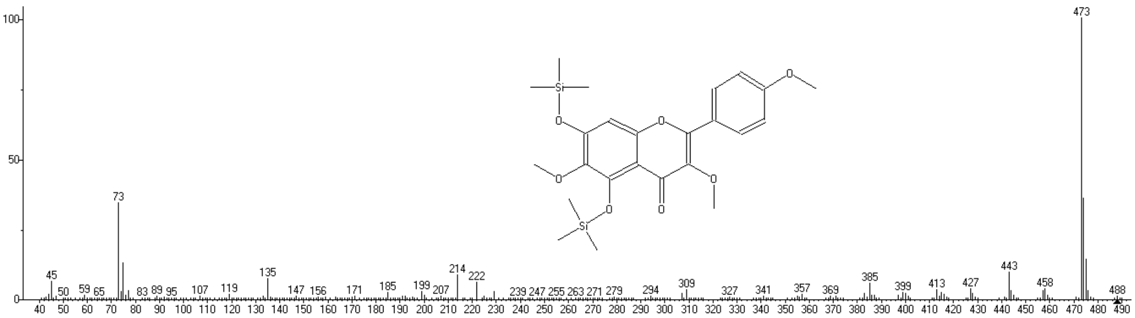

Figure S2. Mass spectra of flavonoids quantified in extracts from birch buds. 1 - TMS derivative of naringenin-7,4'-*O*-dimethyl ether, 2 - di-TMS derivative of sakuranetin, 3 - di-TMS derivative of kumatakenin, 4 - di-TMS derivative of cirsimaritin, 5 - di-TMS derivative of ermanin, 6 - di-TMS derivative of santin.

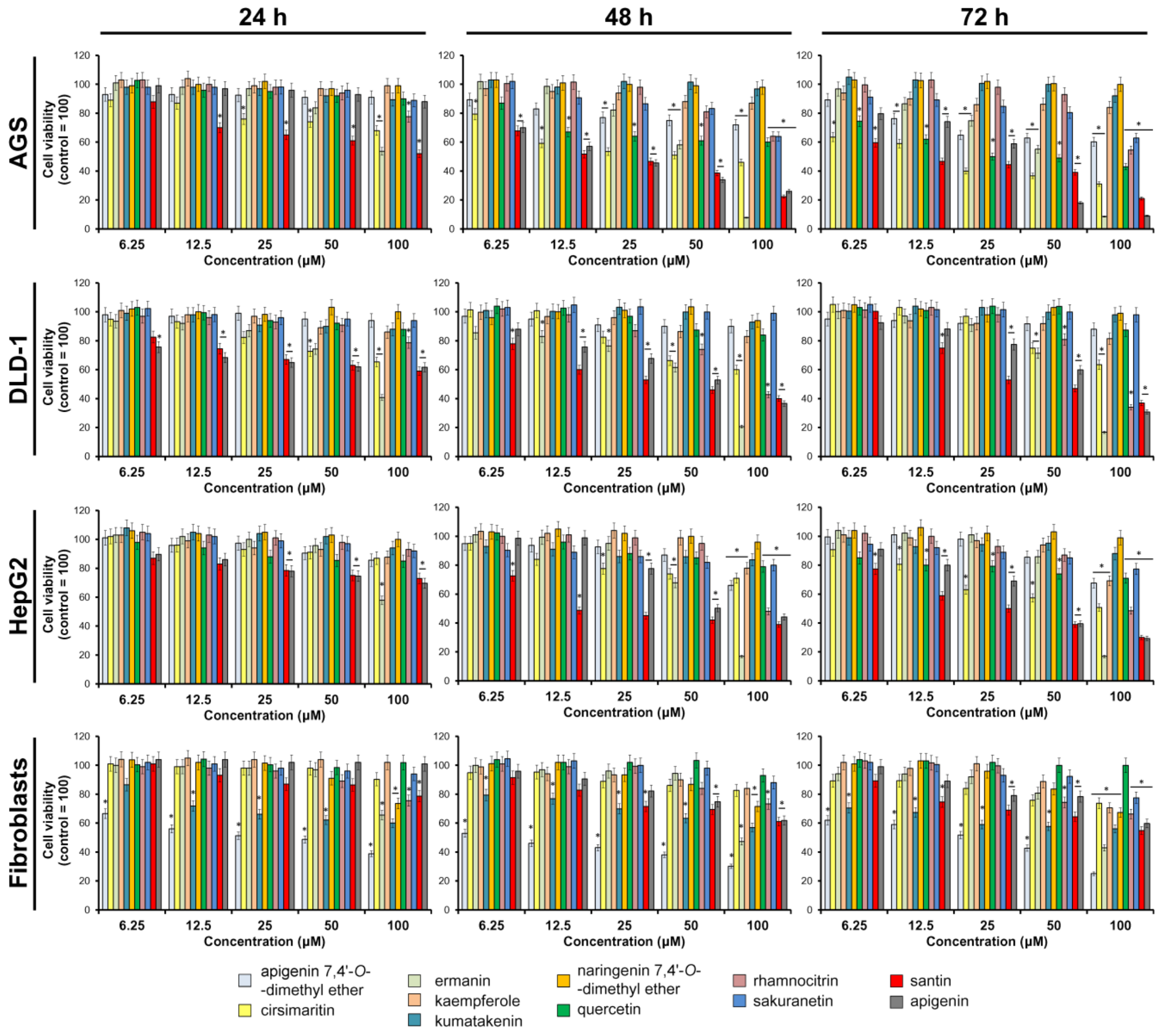

Figure S3. Flavonoids from birch buds differentially affect viability of digestive system cancer cells and fibroblasts. Gastric adenocarcinoma AGS cells, colorectal adenocarcinoma DLD-1 cells, hepatocellular carcinoma HepG2 cells and normal human skin CCD25Sk fibroblasts were treated with different doses of flavonoids for 24, 48 and 72 h. Cell viability was determined using the MTT assay. 0.1% DMSO was used as a vehicle control. Data were expressed as mean ± SD (n = 3). The asterisk indicates $P < 0.05$.

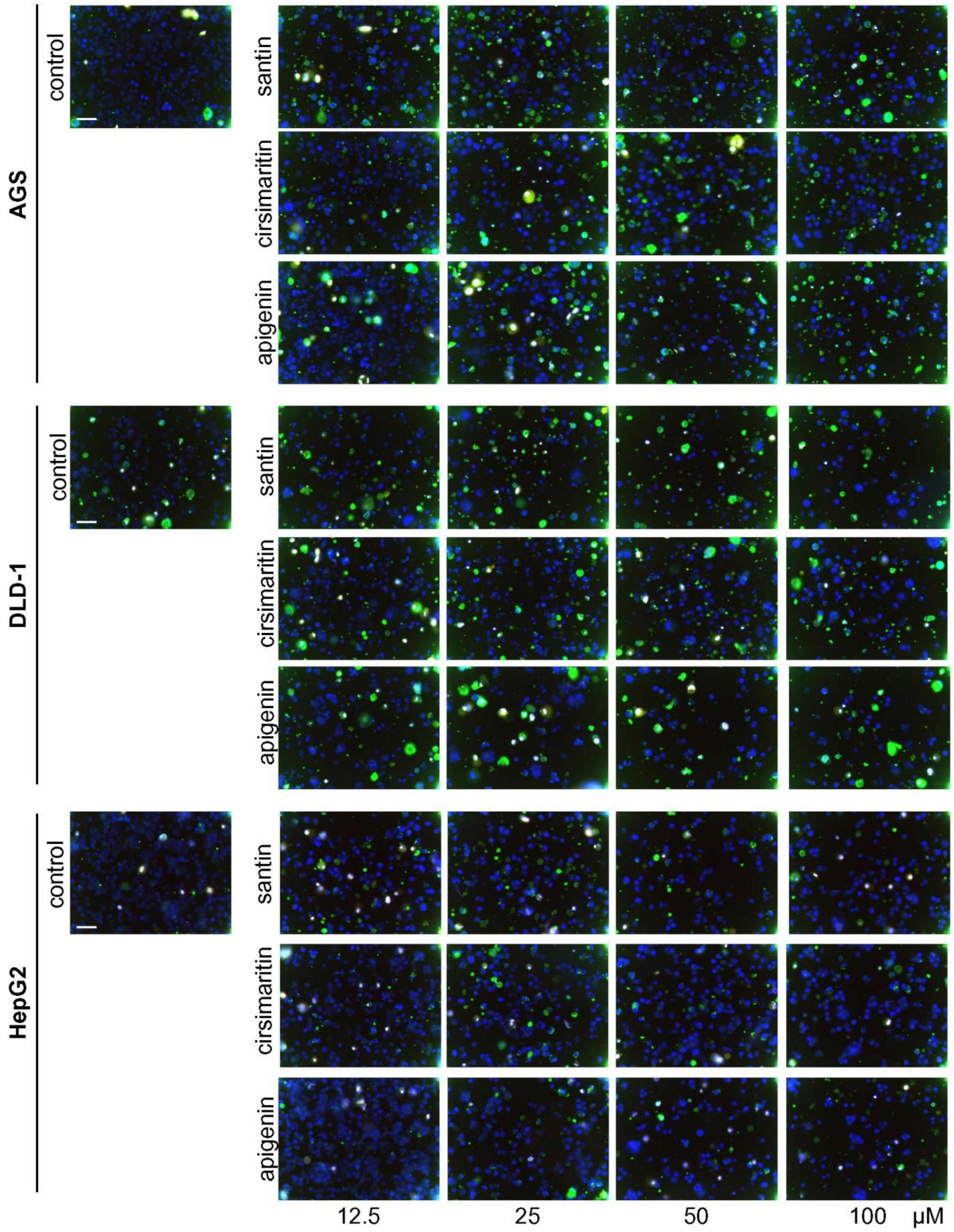

Figure S4. Representative merged photographs of cells stained with annexin V-FITC conjugate (green fluorescence), propidium iodide (red fluorescence), and Hoechst 33342 (blue fluorescence), visualised by fluorescence microscopy. Scale bar = 50 µm.

Table S1. Ions (m/z = M^+-15) used for quantification of individual flavonoids.

Flavonoid	m/z = M^+ - 15
Naringenin-7,4'-*O*-dimethyl ether, TMS	357
Sakuranetin, di-TMS	415
Kumatakenin, di-TMS<br>Cirsimaritin, di-TMS<br>Ermanin, di-TMS	443
Santin, di-TMS	473

Full-length blots

AGS cells

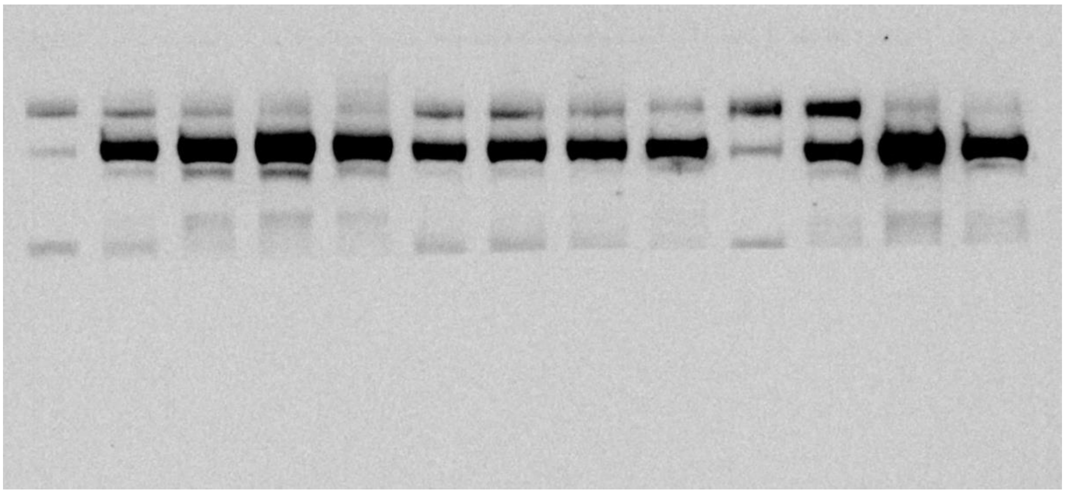

PARP

cPARP

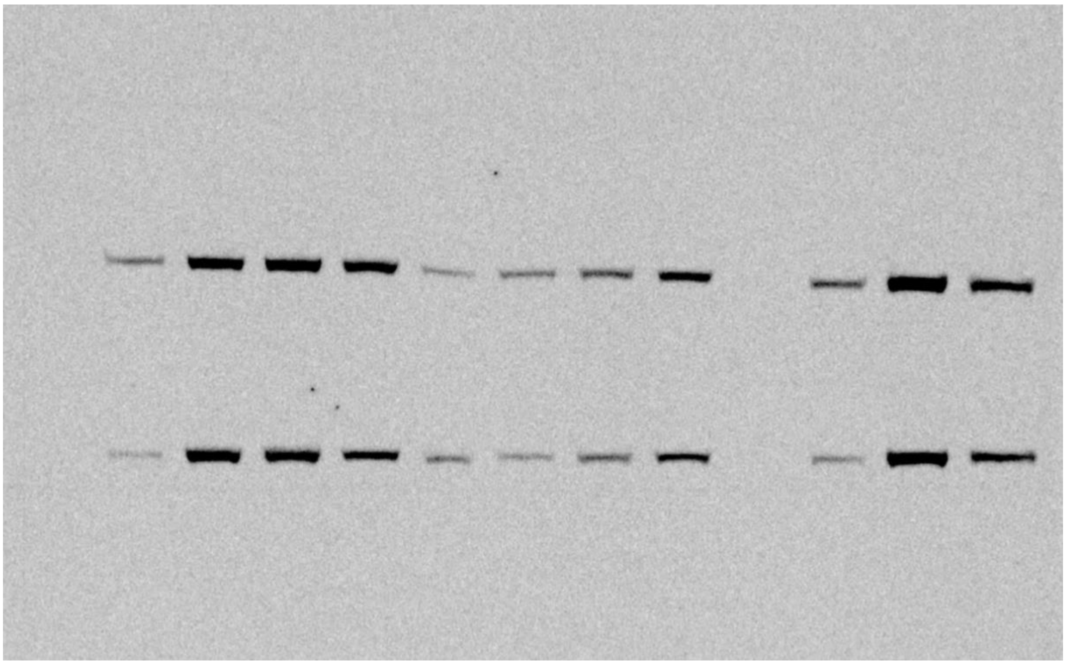

cPARP

(blot presented

in figure)

cPARP

(duplicate)

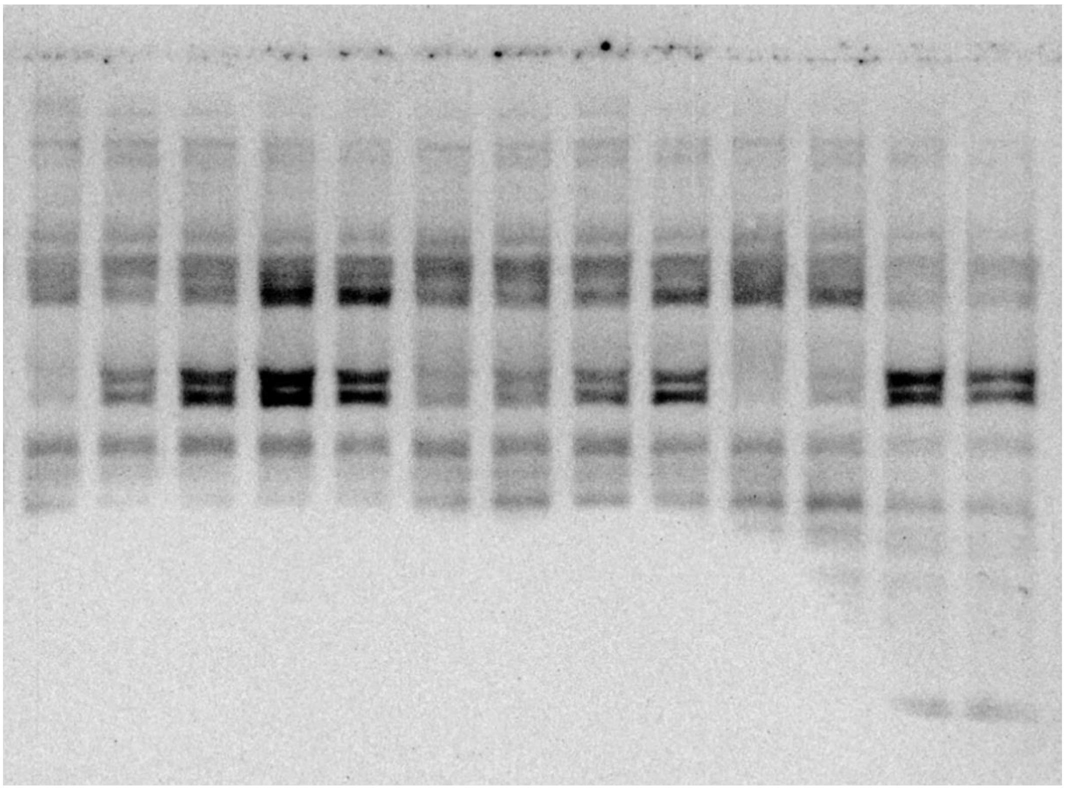

caspase-8

cleaved caspase-8

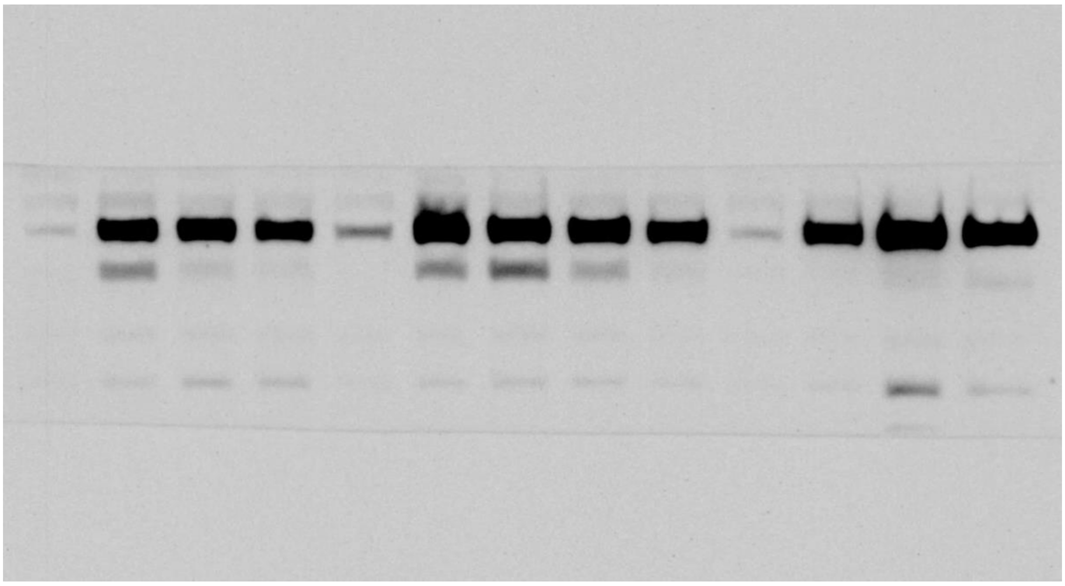

p53

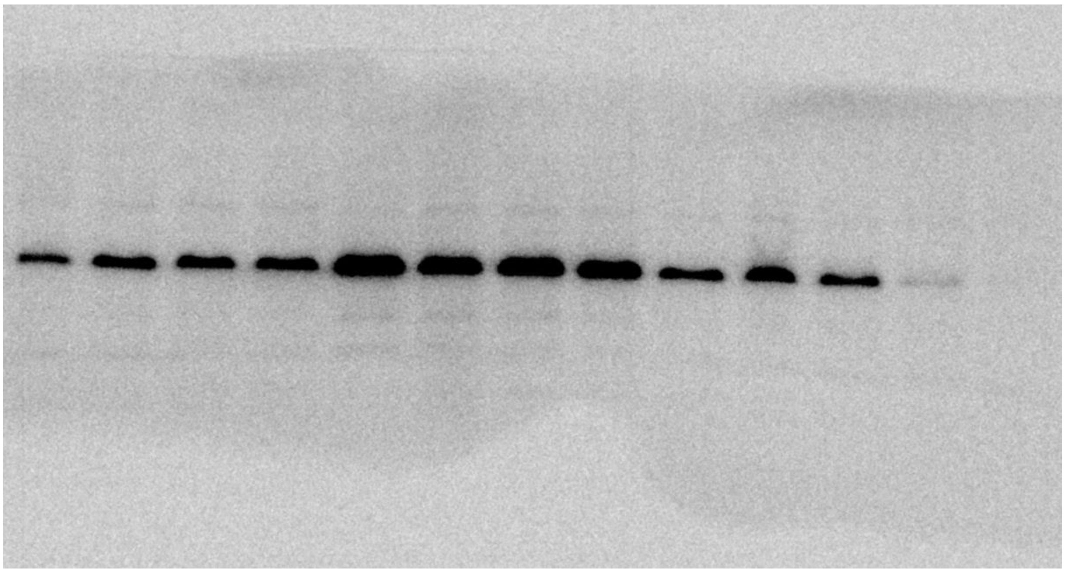

PUMA

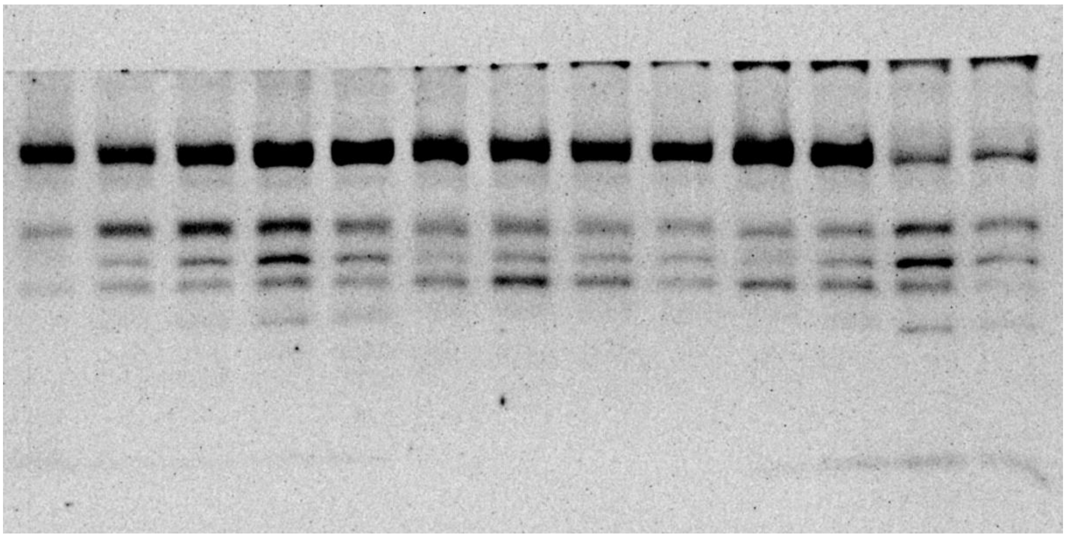

caspase-9

cleaved caspase-9

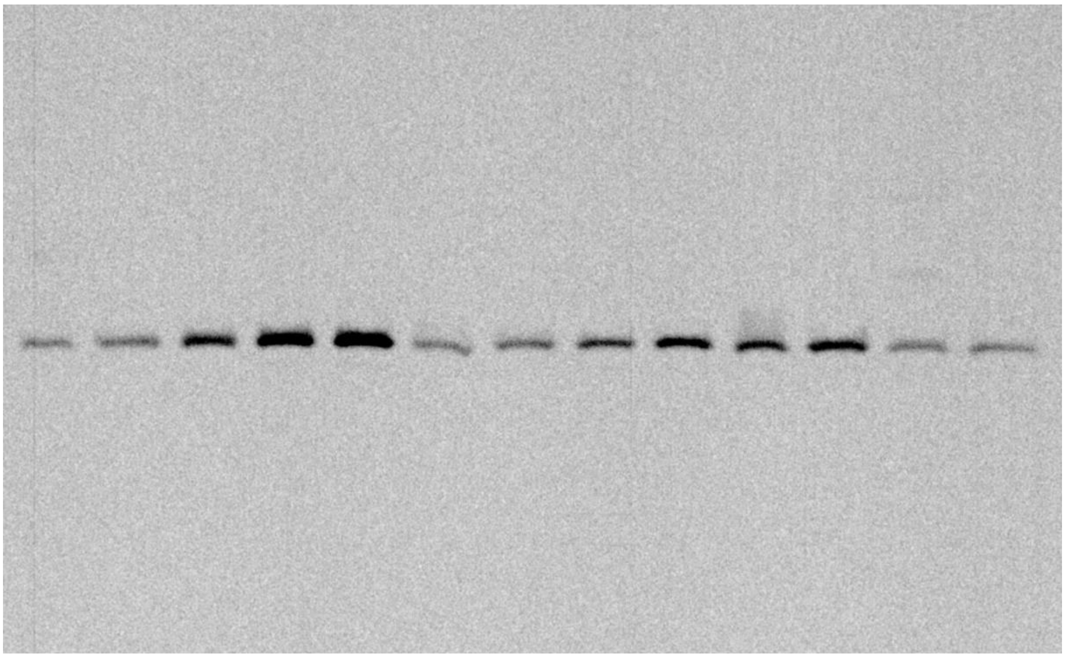

caspase-3

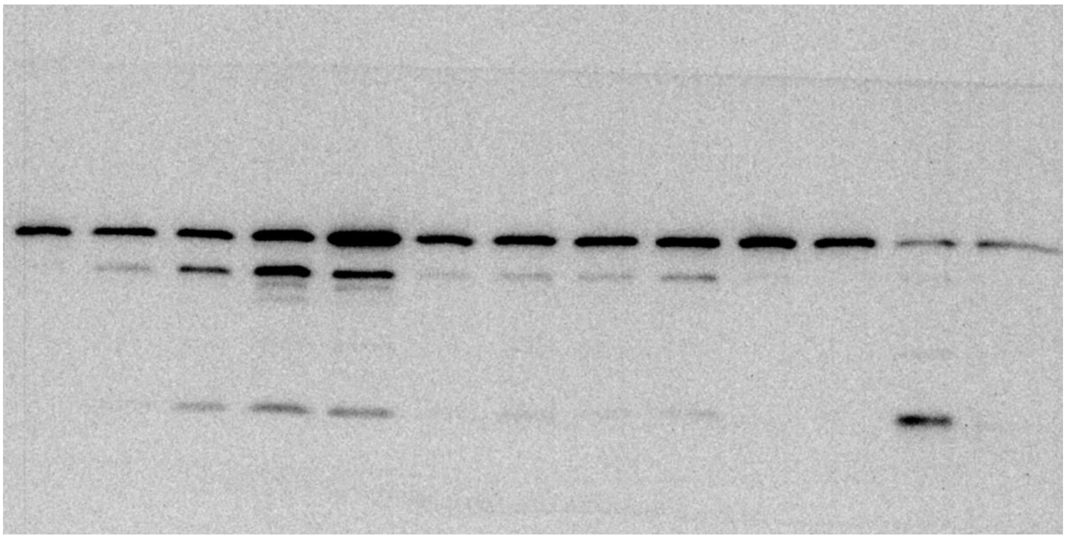

caspase-7

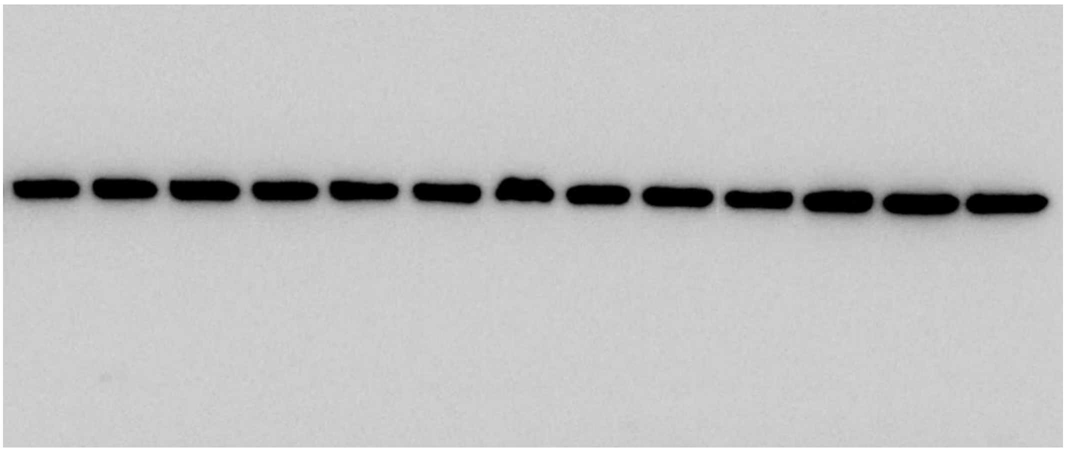

actin

HepG2 cells

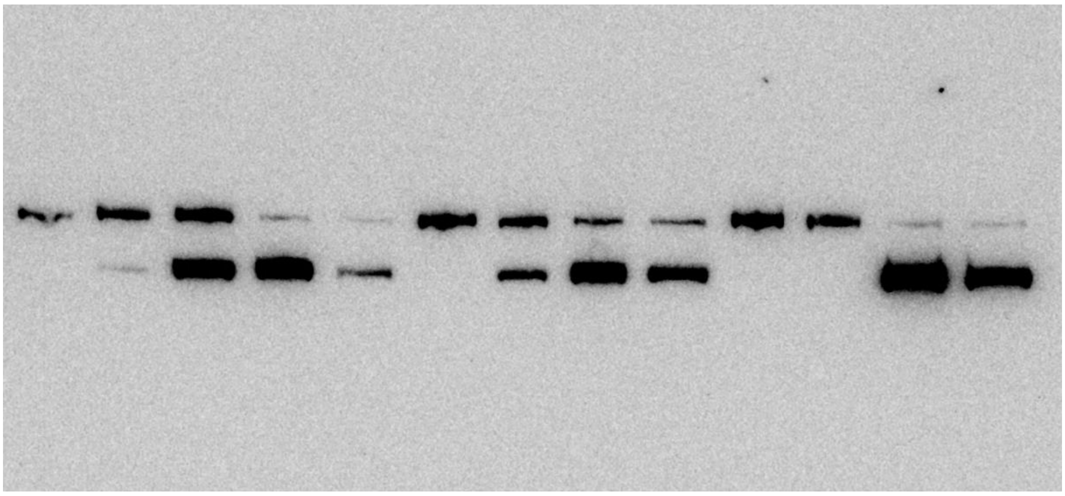

PARP

cPARP

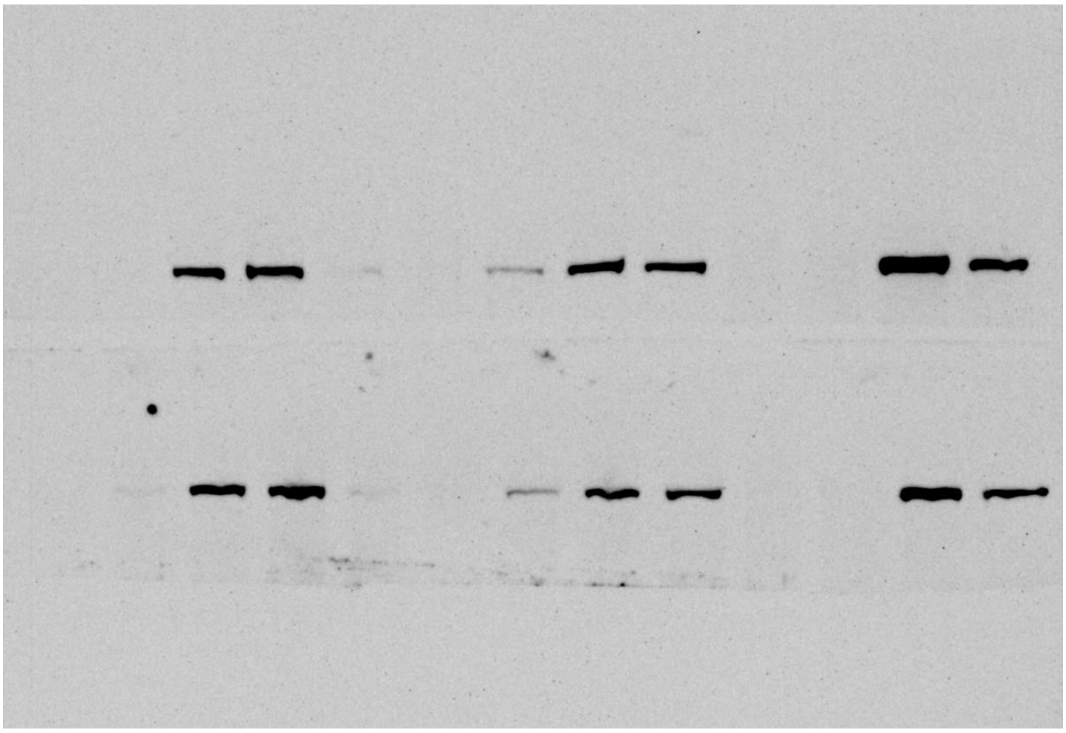

cPARP
(blot presented
in figure)

cPARP
(duplicate)

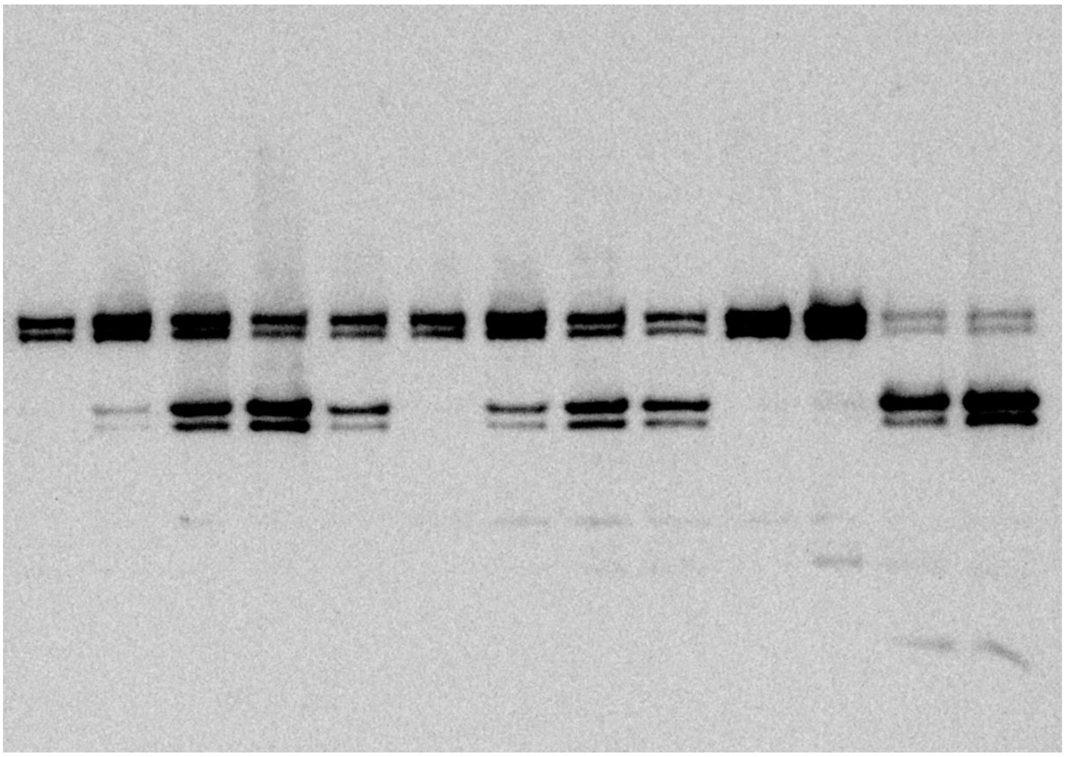

caspase-8

cleaved caspase-8

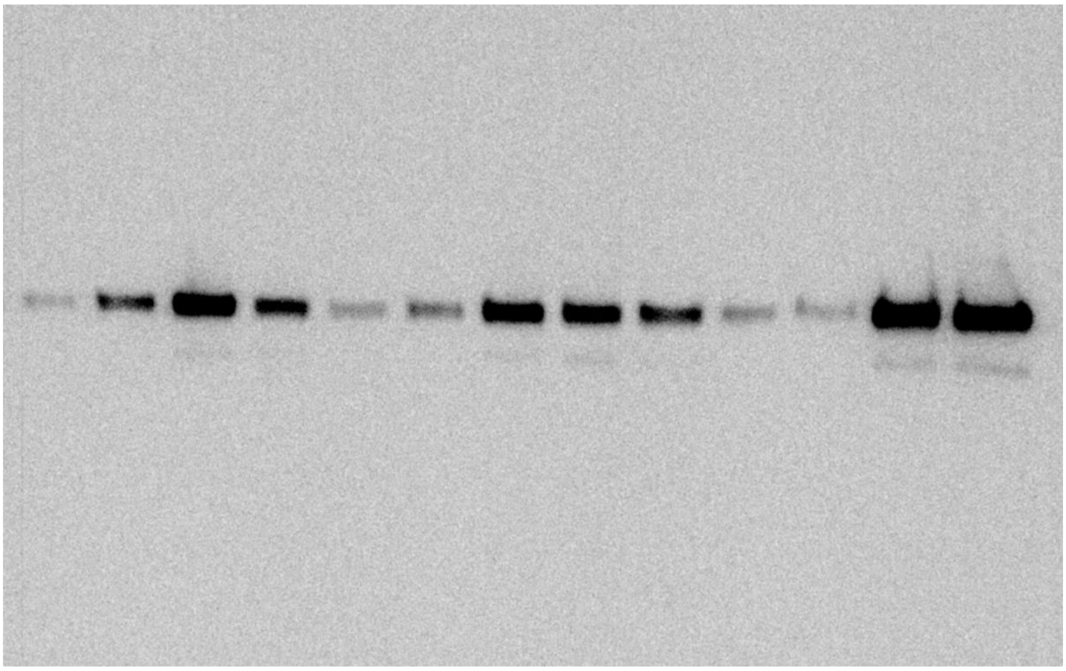

p53

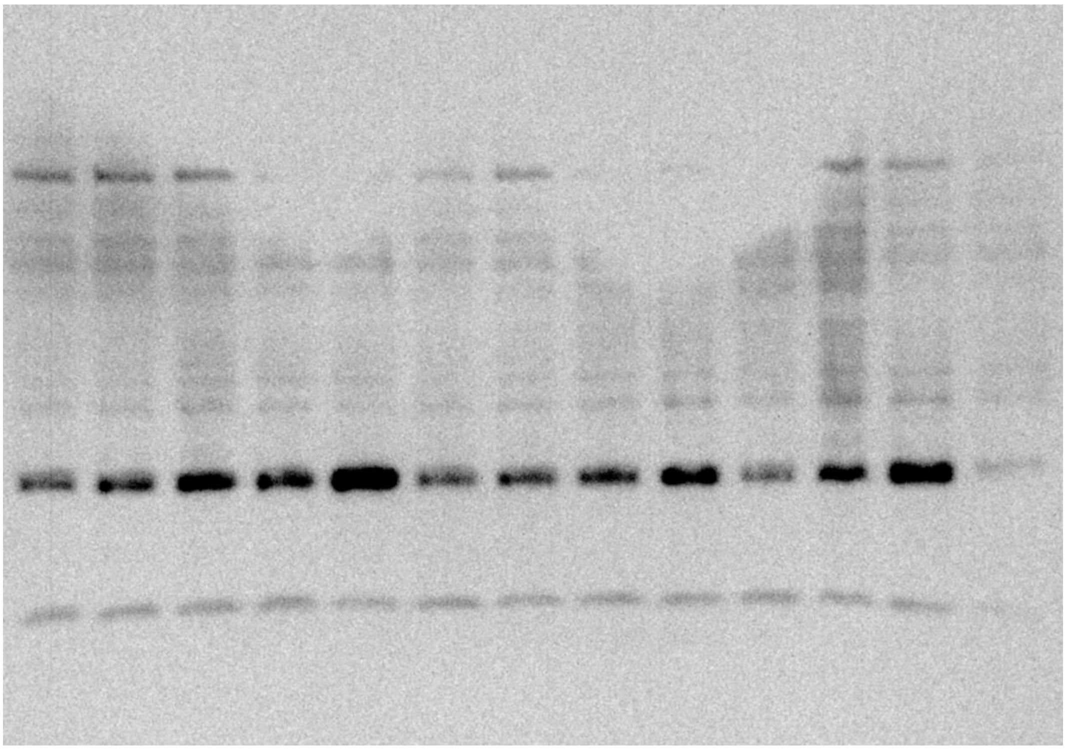

PUMA

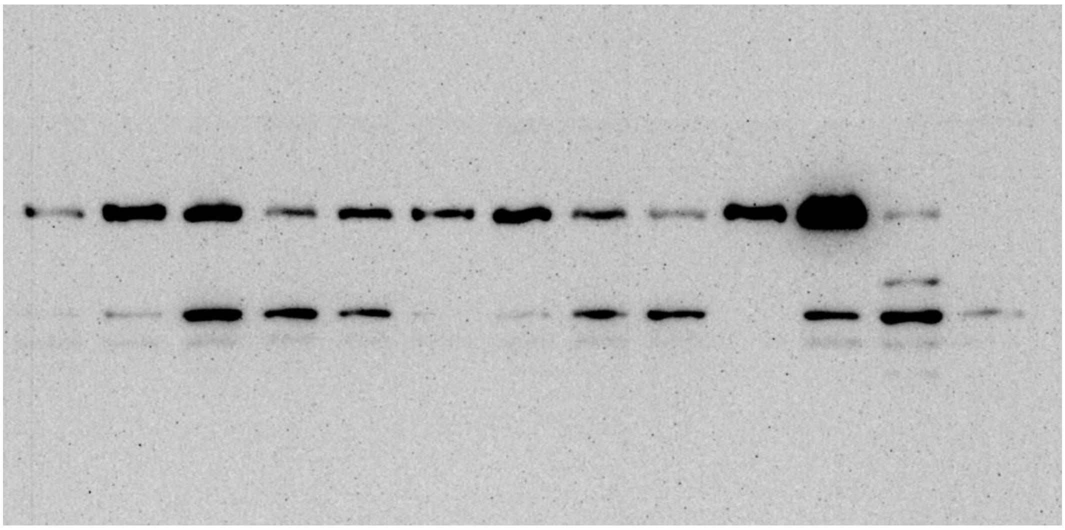

caspase-9

cleaved caspase-9

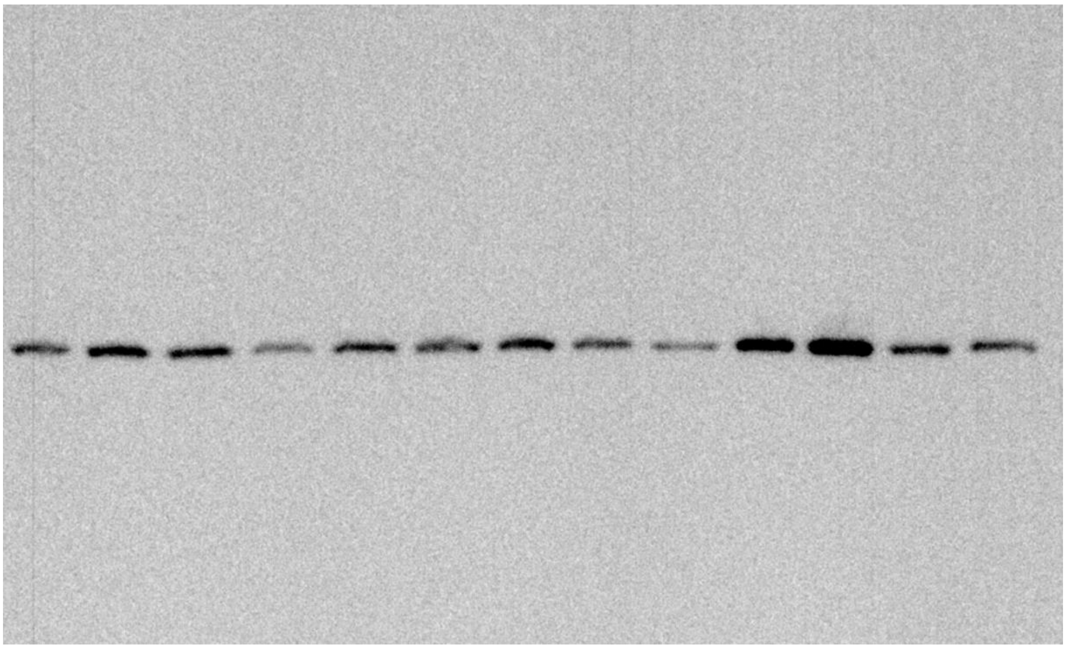

caspase-3

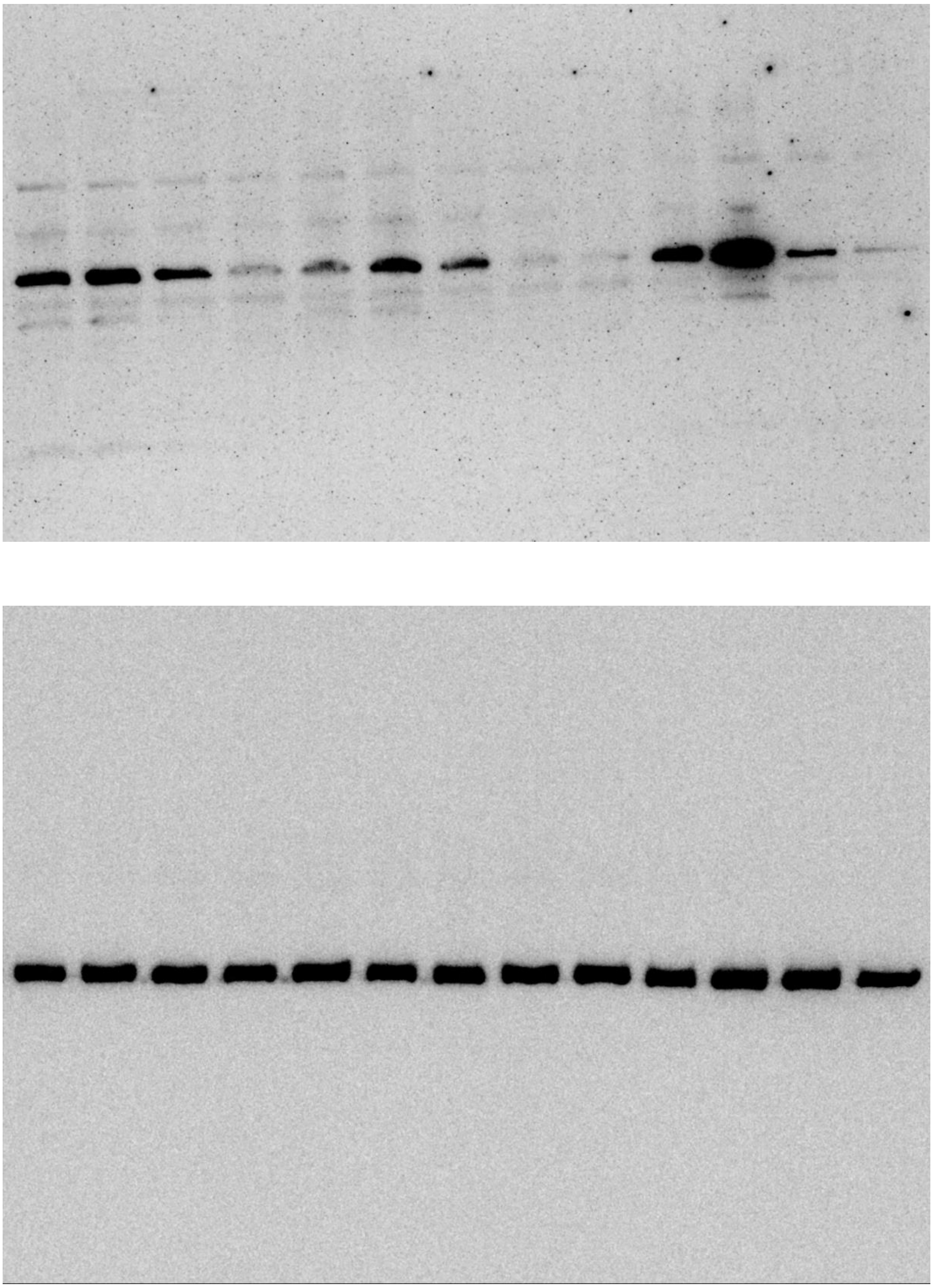

caspase-7

actin

DLD-1 cells

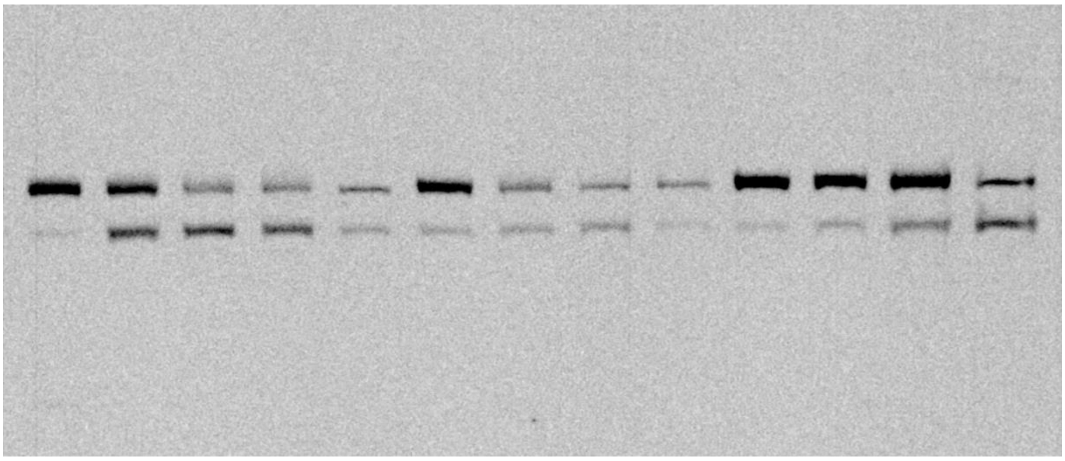

PARP
cPARP

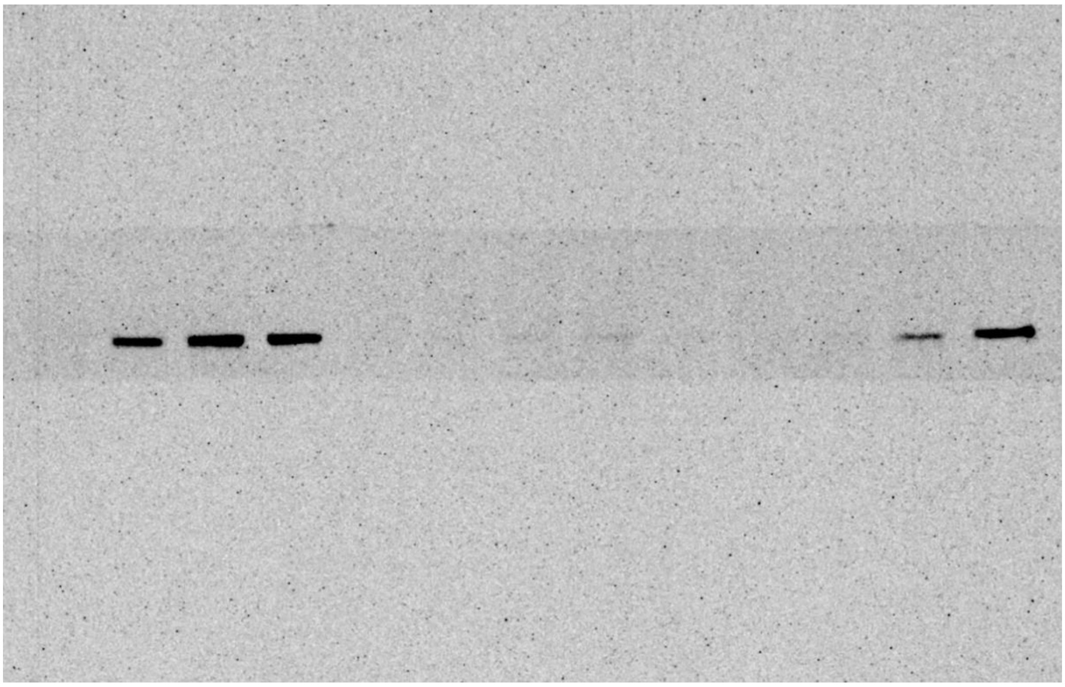

cPARP

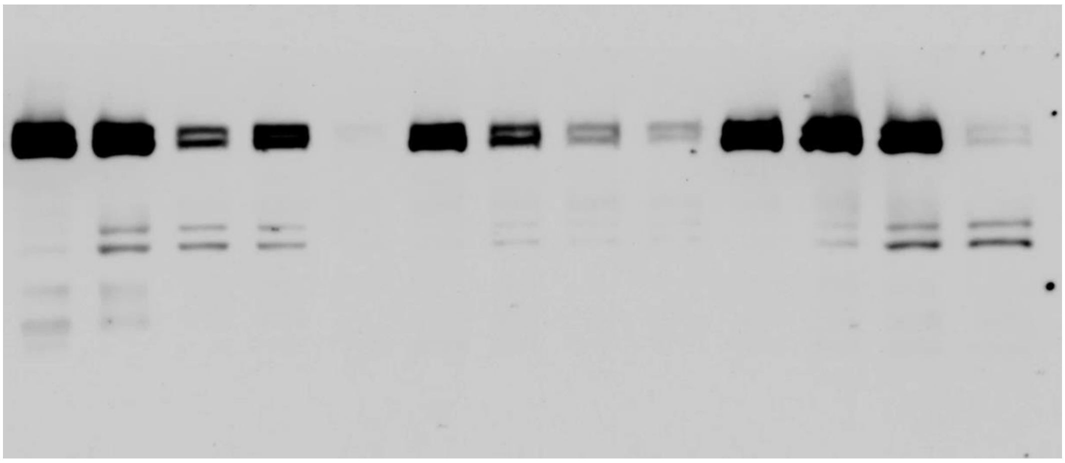

caspase-8
cleaved caspase-8

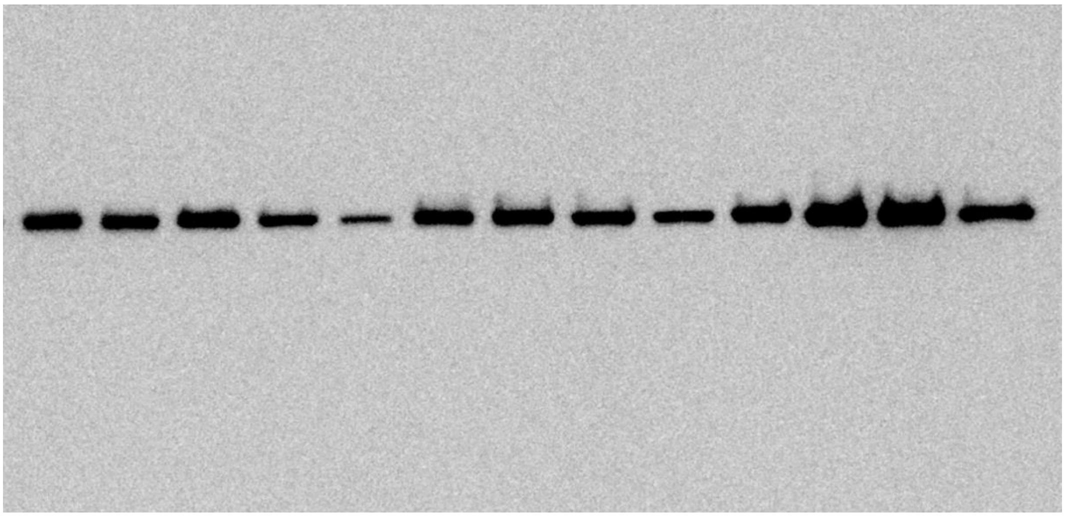

p53

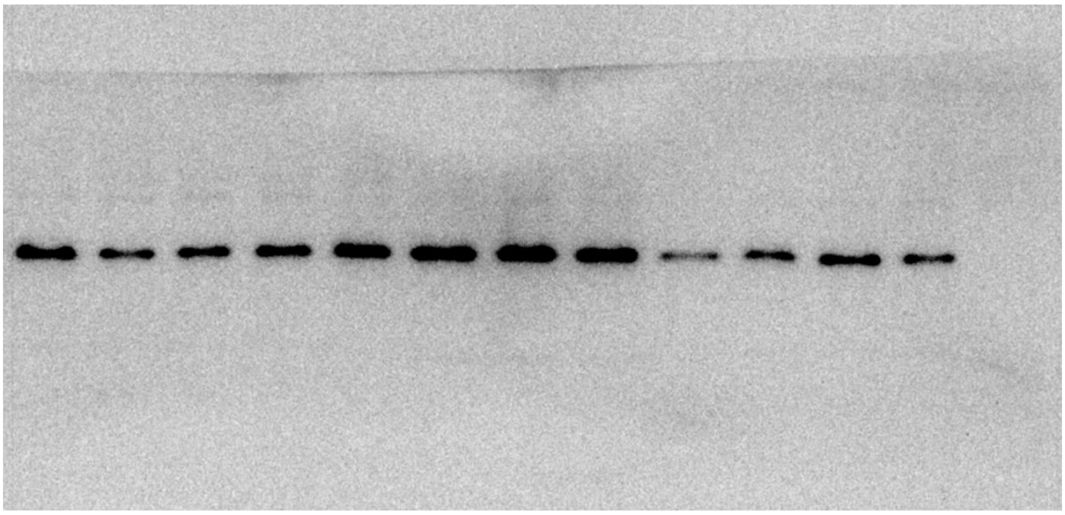

PUMA

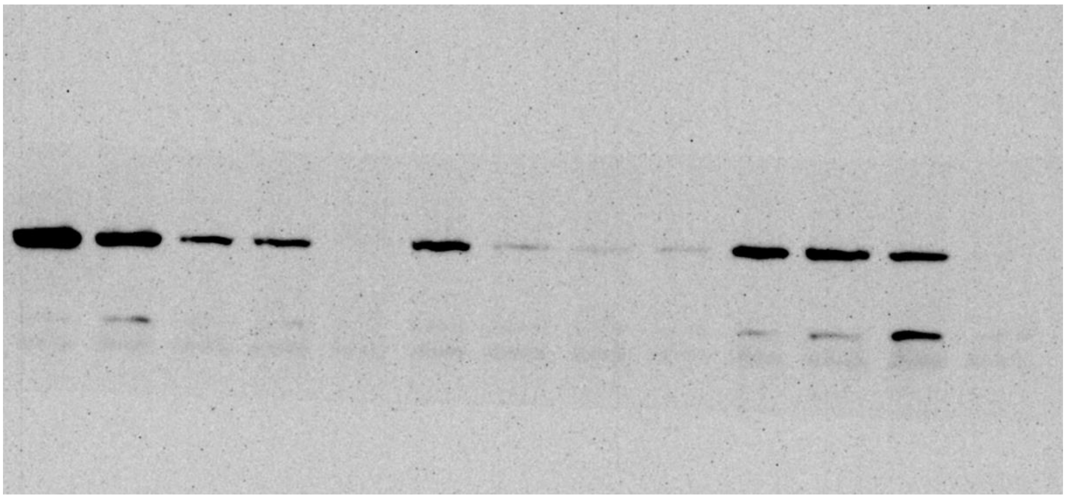

caspase-9

cleaved caspase-9

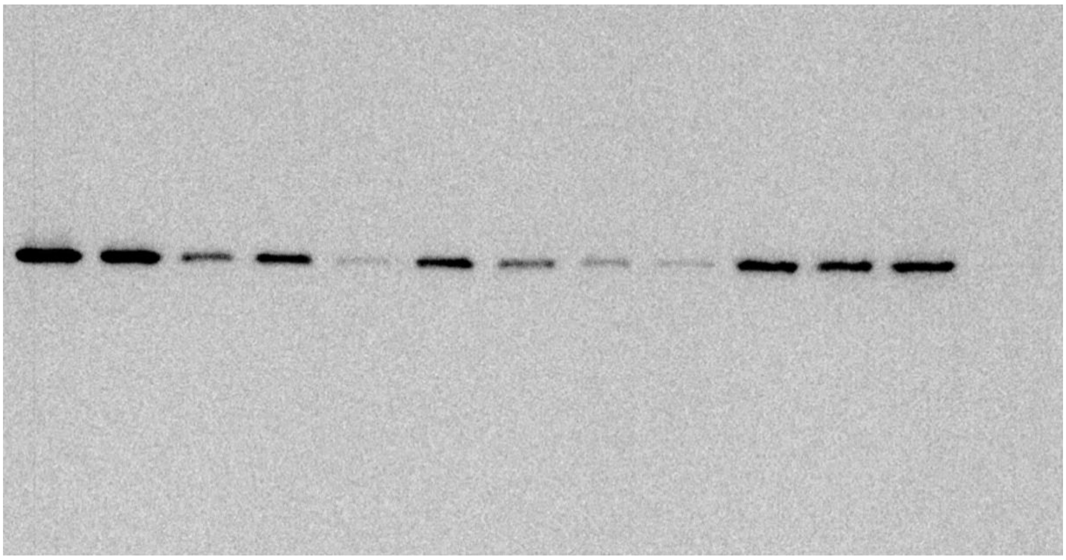

caspase-3

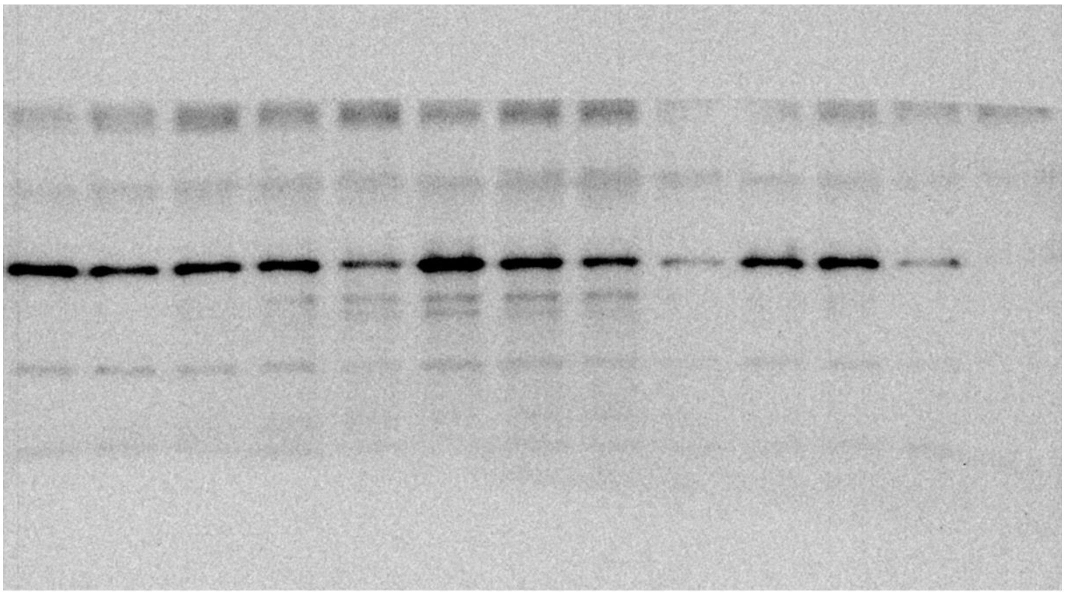

caspase-7

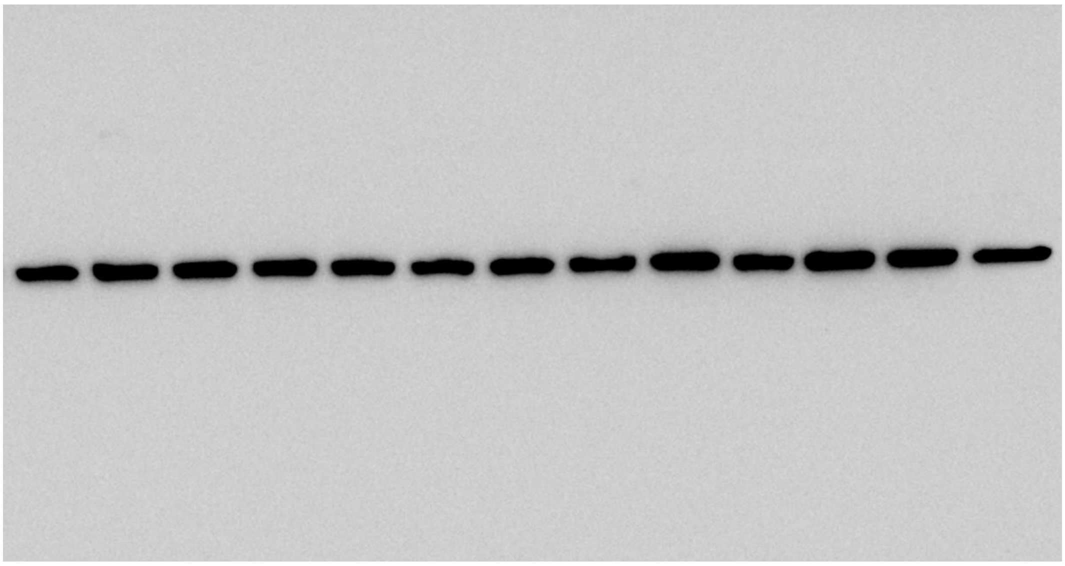

actin
